# Identification and Characterization of MicroRNAs in Small Brown Planthopper (*Laodephax striatellus*) by Next-Generation Sequencing

**DOI:** 10.1371/journal.pone.0103041

**Published:** 2014-07-24

**Authors:** Guoyan Zhou, Tingzhang Wang, Yonggen Lou, Jia'an Cheng, Hengmu Zhang, Jian-Hong Xu

**Affiliations:** 1 College of Agriculture & Biotechnology, Zhejiang University, Hangzhou, China; 2 Institute of Virology and Biotechnology, Zhejiang Academy of Agricultural Sciences, Hangzhou, China; University of Nevada School of Medicine, United States of America

## Abstract

MicroRNAs (miRNAs) are endogenous non-coding small RNAs that regulate gene expression at the post-transcriptional level and are thought to play critical roles in many metabolic activities in eukaryotes. The small brown planthopper (*Laodephax striatellus* Fallén), one of the most destructive agricultural pests, causes great damage to crops including rice, wheat, and maize. However, information about the genome of *L. striatellus* is limited. In this study, a small RNA library was constructed from a mixed *L. striatellus* population and sequenced by Solexa sequencing technology. A total of 501 mature miRNAs were identified, including 227 conserved and 274 novel miRNAs belonging to 125 and 250 families, respectively. Sixty-nine conserved miRNAs that are included in 38 families are predicted to have an RNA secondary structure typically found in miRNAs. Many miRNAs were validated by stem-loop RT-PCR. Comparison with the miRNAs in 84 animal species from miRBase showed that the conserved miRNA families we identified are highly conserved in the Arthropoda phylum. Furthermore, miRanda predicted 2701 target genes for 378 miRNAs, which could be categorized into 52 functional groups annotated by gene ontology. The function of miRNA target genes was found to be very similar between conserved and novel miRNAs. This study of miRNAs in *L. striatellus* will provide new information and enhance the understanding of the role of miRNAs in the regulation of *L. striatellus* metabolism and development.

## Introduction

Small RNAs (sRNAs) including microRNAs (miRNAs), small interfering RNAs (siRNAs), and piwi-interacting RNAs (piRNAs) have been recognized as an important class of gene expression regulators [1]–[5]. miRNAs have an important function to regulate gene expression through sequence-specific base-pairing with their targets in eukaryotes [6], [7], specifically by binding to the 3′ untranslated region (3′UTR) of their target mRNAs through complete sequence complementarity via the ‘seed’ region from positions 2 to 8 in the animal kingdom [2], [8], [9]. miRNAs are involved in almost all physiological processes, including developmental timing, cell division and differentiation, cell proliferation and death, metabolic control, transposon silencing, and antiviral defense [10], [11]. It has been suggested that the regulation of gene expression by miRNAs is very complex, because a single miRNA can regulate hundreds of target genes, and a single gene can be targeted by multiple miRNAs simultaneously [12].

Insects are a group of living creatures that have a huge species diversity, a broad-range ecological niche, and a long evolutionary history. Insects play important roles in human life and the function of environmental ecology. Three major rice planthoppers belonging to Hemiptera: Delphacidae are considered the most notorious crop pests within Asia: small brown planthopper (*Laodephax striatellus* Fallén), brown planthopper (*Nilaparvata lugens* Stål), and white-backed planthopper (*Sogatella furcifera* Horvath) [13]–[15]. *N. lugens* and *S. furcifera* can cause loss of rice yield by sucking the phloem sap of rice, whereas *L. striatellus* can cause great damage to crops not only by direct feeding but also by transmitting plant viruses, such as rice stripe virus, rice black-streaked dwarf virus, maize rough dwarf virus, and wheat rosette virus [14], [16], [17]. Previous studies have mainly focused on the entomology, population biology, plant protection, and viruses involved with these insects, due to the fact that the whole genomes of the rice planthoppers have not been sequenced [18]–[20]. After the first miRNA, *lin-4*, was discovered in nematode worms 20 years ago [21], miRNAs have been identified in worms, flies, mammals, higher plants, and even unicellular algae [22]–[24]. A large number of miRNAs have been identified through the use of next-generation sequencing (NGS) technology. These miRNAs included about 3456 miRNAs from various insect species and have been deposited into miRBase, Release 20 [12], [25]–[33].

Recently, sRNA and transcriptome data have been made available for *N. lugens* [15], [25], but the genome sequence information for *L. striatellus* has not been fully researched. In this study, a sRNA library was constructed from mixed developmental stages of *L. striatellus* and sequenced by Solexa sequencing technology. This process allowed for the identification of 501 mature miRNAs. Most of the miRNA families that are considered conserved among various insect species are highly conserved in the Arthropoda phylum, and the functions of the putative target genes are very similar between conserved and novel miRNAs. Our results will provide new information and the further understanding of miRNAs in the regulation of gene expression in *L. striatellus* metabolism and development.

## Results and Discussion

### Deep sequencing and sRNA analysis

**Figure 1:**
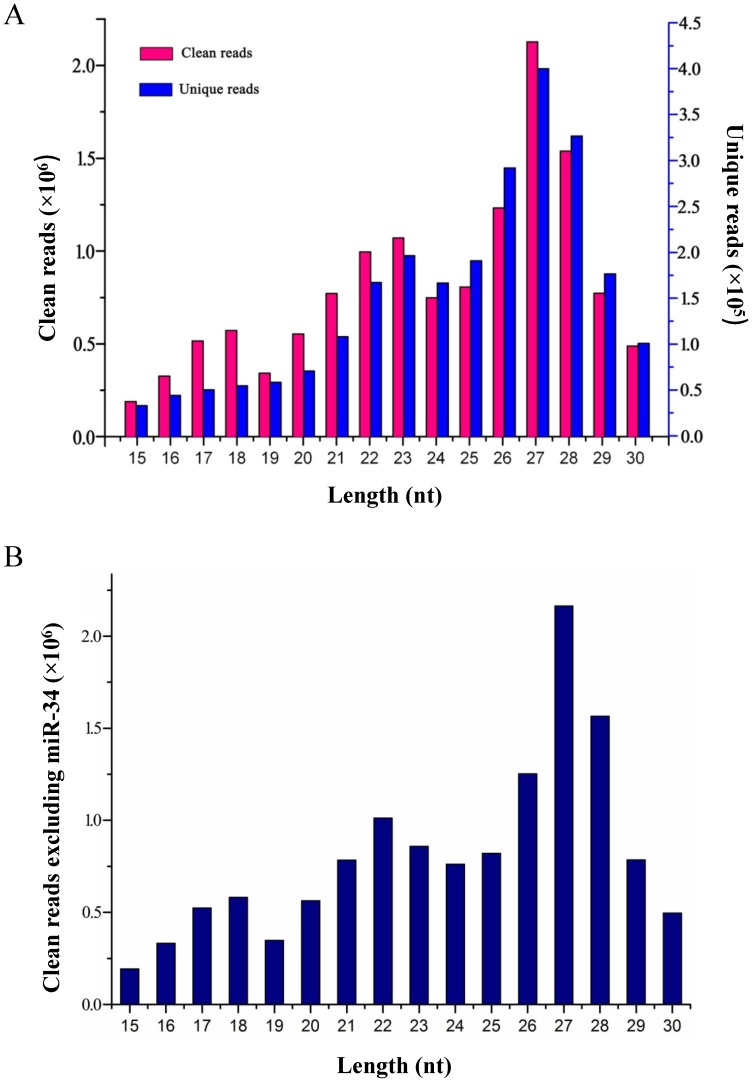
Length distribution of sRNAs in *L. striatellus*. (A) The total number of clean and unique reads of sRNAs 15 to 30 nt in length; (B) the clean reads that excluded miR-34.

Total RNA was extracted from a mixed *L. striatellus* population, and a sRNA library was constructed and sequenced using Illumina Solexa sequencing technology. A total number of 18,510,874 raw reads were obtained, and 13,275,597 clean reads and 2,464,934 unique reads of 15 to 30 nucleotides (nt) in length remained for further analysis after discarding the reads of incorrectly indexed sequences, low-quality sequences, sequences without 3′ adaptors, sequences longer than 30 nt and shorter than 15 nt, and simple-repeat sequences (Table 1). Most of these sequenced sRNAs were found to be around 27 nt in length. The next group of sRNAs were about 28 nt and 26 nt in both clean reads and unique reads. The last group of sRNAs was found to have two peaks: one peak at 23 nt and the second peak at 18 nt, respectively (Figure 1A).

**Table 1:** Summary of sRNA sequencing data.

Category	Total reads	Percentage of total reads (%)
Raw data	18,510,874	100
Incorrectly indexed reads	342,546	1.85
Low-quality reads	320,233	1.73
3′ adaptor null reads	315,308	1.70
Short reads	1,263,815	6.83
Simple-repeat reads	693	0.004
Long reads	2,992,682	16.17
Clean reads	13,275,597	71.72
Unique reads	2,464,934	

Previous work showed that silkworm, bee, locust, purple urchin, and Lancelet fishes have sRNA peak lengths at 22 nt and 18 nt [12], [27], [32], [34], [35], suggesting that 22-nt sRNAs are the most abundant sRNAs within the animal kingdom. sRNAs in zebrafish at early developmental stages were shown to have three peaks of length 18 nt, 23 nt, and 27 nt, which were found to shift to 18 nt, 22 nt, and 27 nt in later developmental stages [36]. In butterflies, the sRNAs showed a peak at 23 nt, which was caused by high reads of miR-31. After the miR-31 reads were excluded, the sRNA peak at 23 nt shifted to 22 nt [37]. Interestingly, in our study, when the reads of the most abundant miRNA (miR-34) were excluded, the sRNA reads had three peaks of the following lengths: 18 nt, 22 nt, and 27 nt. This is consistent with the sRNA peaks observed in other animals (Figure 1B).

In many studies, the sRNA peak of 18 nt is attributed to tRNA-derived sRNAs and rRNA-related sRNAs, whereas an sRNA peak of 27-nt is considered to be derived primarily from repetitive sequences and/or piRNAs [36], [37]. In *N. lugens*, the highest number of sRNAs reads using a set of three sRNA libraries that consisted of female adult, male adult, and the last instar female nymph was found to be 27 nt, 22 nt, and 27 nt, respectively [25]. This suggests that the majority of sRNAs in female *N. lugens* are 27 nt in length. Likewise, the majority of sRNAs in our library were found to be 27 nt, which were derived from a mixed group of *L. striatellus* at various developmental stages that likely included more females than males.

**Figure 2:**
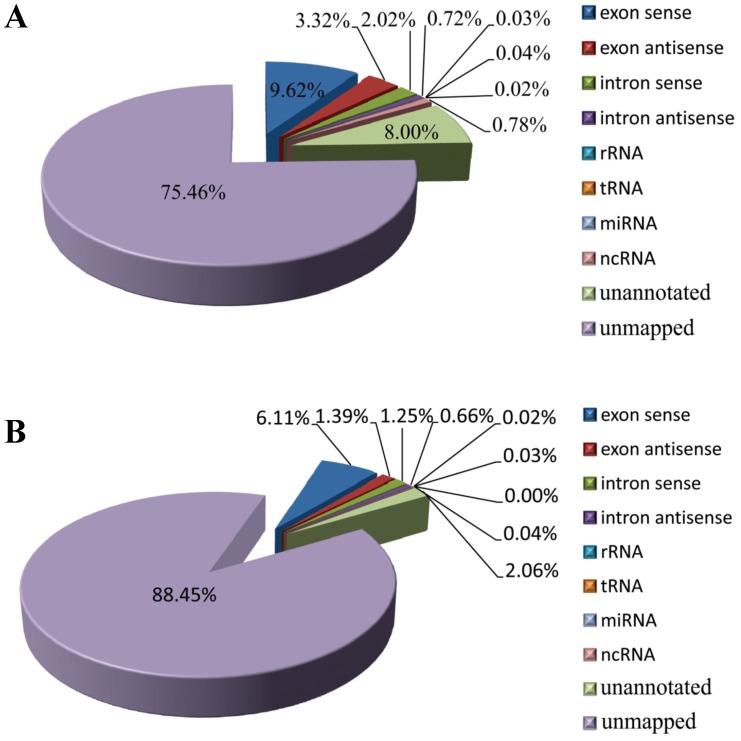
sRNA annotations of *L. striatellus*. (A) Total sRNA annotations; (B) unique sRNA annotations.

All clean reads were mapped on the *D. melanogaster* genome (http://www.fruitfly.org/) using Bowtie software. The majority of sRNAs were unmapped, whereas 10–15% of sRNAs were located within genes (in exons or introns), less than 1% were identified as rRNA, tRNA, miRNA, or snRNA, and the remaining were unannotated (Figure 2A, 2B). A previous study revealed that the mitochondrial genome was found to encode abundant small non-coding RNAs (mitosRNAs) [38]. All clean reads were then mapped on the mitochondrial genome of *L. striatellus* [39]. A total of 3977 unique reads were mapped to mRNAs (1546), tRNAs (308), and rRNAs (2091). This suggests that the mitochondrial genome encodes mitosRNAs in animals. However, the mapped *L. striatellus* sRNAs had peaks at 22 nt (unique reads) and 23 nt (redundant reads) (Figure S1), which was different from the peak at 30 nt observed in the human and mouse genomes [38]. Thus, it can be concluded that different mechanisms may be used to produce mitosRNAs in different species [38].

### Identification of conserved and novel miRNAs

**Figure 3:**
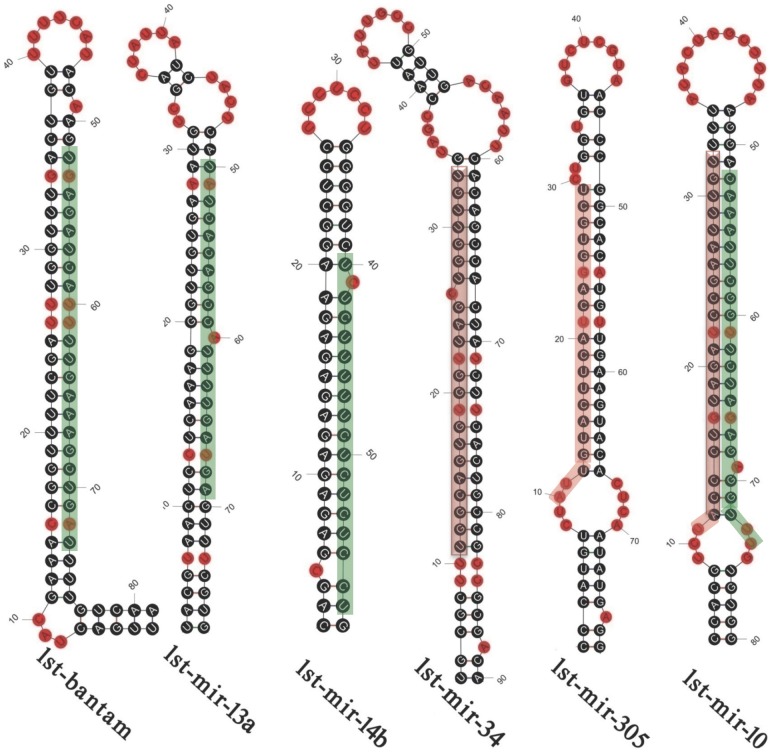
Secondary structures of six representative miRNA precursors in *L. striatellus*. A green bar indicates the mature miRNA located in the 3' end, whereas a pink bar represents the mature miRNA located in the 5' end. Red circles indicate mismatches, and black circles show complementary base pairing.

To identify conserved miRNAs in our data, around 24% total and 11.5% unique clean sRNA reads, which included gene-related sRNAs, known miRNAs, and unannotated sRNAs, were aligned with the mature miRNAs and precursors in miRBase 20.0 (http://www.mirbase.org/), and miRNAs from *N. lugens* [25] with no mismatches or having only one mismatch. We identified 219 conserved miRNAs belonging to 125 miRNA families (Table S1). miRNA is derived from a ∼70-nt stem-loop precursor miRNA, originating from a single-stranded RNA transcript through successive endonucleolytic cleavages by Drosha or DGCR8/Pasha proteins in animals [40]. The miRNA precursor has a representative hairpin structure, which is the primary criterion used to identify mature miRNA [41]. We then aligned all 219 miRNA sequences to the *D. melanogaster* genome, *N. lugens* transcriptome [15], and the mitochondrial genome of *L. striatellus* [39]. Interestingly, we obtained eight miRNAs derived from 16 precursors (each with two different precursors) and 53 miRNAs derived from 37 precursors that were predicted to have the representative hairpin structures, which fit all miRNA filter criteria (See Materials and Methods for details). We obtained a total of 69 mature miRNAs that were derived from their precursors with hairpin structures and fit all miRNA filter criteria. The hairpin structures of six representative conserved miRNAs are shown in Figure 3, and detailed information and hairpin structures of all miRNAs are listed in Table S2 and Figure S2. All of these conserved miRNAs belong to 38 miRNA families (Table S3). However, the stem-loop structure precursors of most conserved miRNAs (158) could not be found, which may be attributed to the absence of the whole-genome data for *L. striatellus*.

**Figure 4:**
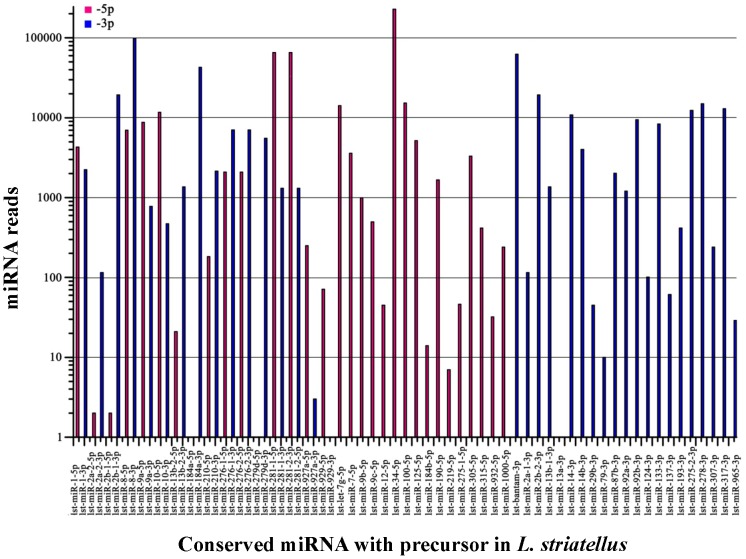
The abundance of conserved miRNAs in *L. striatellus*. The number of miRNA reads is shown for precursor miRNAs with both 5′ and 3′ mature miRNAs, those with only 5′ mature miRNAs, and those with only 3′ mature miRNAs.

All conserved miRNAs with stem-loop structures can be divided into three types: (1) 32 conserved miRNAs that represent the 5′ and 3′ mature miRNAs of 16 precursor miRNAs, and both mature miRNAs are conserved; (2) 16 conserved miRNAs that represent only the 5′ mature miRNAs; and (3) 21 miRNAs that represent only the 3′ mature miRNAs. The number of reads that aligned to the miRNA was assumed to represent the abundance of the miRNA. The most abundant conserved miRNA identified in the library was lst-miR-34-5p, which had 228,636 reads (Table S3) and accounted for 33% of the total reads of all miRNAs. This miRNA together with four other miRNAs, lst-miR-8-3p, lst-miR-281-1-5p (or lst-miR-281-2-5p), lst-miR-184-3p, and lst-bantam-3p, each with more than 10,000 reads, accounted for 72% of total reads of all miRNAs. More than half of conserved miRNAs (39/69) were abundantly expressed (>1,000 reads). Sixteen miRNAs had fewer than 100 reads; in particular, eight had a very low expression level (<10 reads), and five of the eight were considered to be miRNA*, but high expression was observed for the miRNA derived from the other arm of the same precursor (Figure 4, Table S3). Usually, the expression of miRNA is higher than that of miRNA* [42], [43]. However, it has been observed that many miRNAs* have a much higher expression than miRNAs [44]–[46], and some of them are quite conserved across different species [26], [32]. Therefore, miRNA* may not only maintain the hairpin structure, but this finding suggests a new paradigm for miRNA* function at the post-transcriptional level [44], [47].

The remaining sRNA sequences were aligned to the genome of *D. melanogaster*, the transcriptome of *N. lugens*, and the mitochondrial genome of *L. striatellus* once again to identify potential novel miRNAs. Sequences that met the criteria for miRNA, based on the miRNA prediction software Mireap, were considered to be miRNA precursors. Ultimately, 274 novel miRNAs were identified (Table S2). These included 222 from the *D. melanogaster* genome and 53 from the *N. lugens* transcriptome derived from 268 precursors, which belong to 250 miRNA families based on the criterion that the miRNA family shares the conserved seed region spanning from nucleotides 2 to 8 (Table S3) [48]. One miRNA was found to be present in both the *D. melanogaster* genome and the *N. lugens* transcriptome. Among 250 miRNA families, only 19 families have at least two mature miRNAs (Table 2). Interestingly, ten novel miRNAs were found to have identical mature sequences, but were located at different loci (Table 2).

**Table 2:** The 19 novel miRNA families that have at least two mature miRNAs in *L. striatellus*.

Family	Member	miRNA sequence (5'->3')	Length (nt)	Reads
**miR-n1**	lst-miR-n1-5p	TGAAAAAGTTGGCGTAGA	18	1
	lst-miR-n1-3p	GTGGGCTTCGGCTTTGCCGT	20	66
**miR-n2**	lst-miR-n2-1-5p	TGAACTTGCTGCTGCTGCTGCTGC	24	1
	lst-miR-n2-1-3p^a^	TGCTGGTGTGGCTGCTGGGGG	21	15
	lst-miR-n2-2-3p^a^	TGCTGGTGTGGCTGCTGGGGG	21	15
**miR-n3**	lst-miR-n3-5p	CCTCGTCGTCGTCGAAGATGG	21	1
	lst-miR-n3-3p^a^	ACTTCGAGGAGGAGGAGGACC	21	3
	lst-miR-n77-5p^a^	ACTTCGAGGAGGAGGAGGACC	21	3
**miR-n4**	lst-miR-n4-5p	AGCTGGCTTTAGGGTTTATGGT	22	3
	lst-miR-n4-3p	CCCCGCTCGCCTGCTCCAGC	20	1
**miR-n5**	lst-miR-n5-5p	TTTGAGTTCTTTGAAGTGTGT	21	3
	lst-miR-n5-3p	TTAGGGTTTATGGGATTAG	19	1
**miR-n6**	lst-miR-n6-5p	ACGGCTTATCGTCTTAAAGACA	22	1
	lst-miR-n6-3p	TGGTTGTGGAGTCTGTTGTT	20	4
**miR-n12**	lst-miR-n12-5p	TGGTGGAGGAAATGGTGGAGGGC	23	3
	lst-miR-n190-3p	TGGTGGAGGAGATGGTGGTGGGT	23	2
**miR-n13**	lst-miR-n13-1-5p^a^	TGCTGCTGCTGCTCGAGCTCT	21	3
	lst-miR-n13-2-5p^a^	TGCTGCTGCTGCTCGAGCTCT	21	3
	lst-miR-n198-3p	TGCTGCTGATGATTTTGATTGC	22	7
**miR-n17**	lst-miR-n17-1-5p^a^	CGTCAAAATGGCTGTGAGCT	20	4
	lst-miR-n17-2-5p^a^	CGTCAAAATGGCTGTGAGCT	20	4
**miR-n24**	lst-miR-n24-5p	CGATTCCTGCCCAGGCCACGA	21	53
	lst-miR-n227-3p	CGATTCCATTTGCCTCCGCCA	21	11
**miR-n28**	lst-miR-n28-1-5p^a^	TTGCAGTCGTTGGGCTGGACG	21	4
	lst-miR-n28-2-5p^a^	TTGCAGTCGTTGGGCTGGACG	21	4
**miR-n29**	lst-miR-n29-1-5p^a^	TGAGATGGTTGGATCCTGGC	20	3
	lst-miR-n29-2-5p^a^	TGAGATGGTTGGATCCTGGC	20	3
	lst-miR-n29-3-5p	TGAGATGGTTGGATCCTGGC	20	3
**miR-n37**	lst-miR-n37-1-5p^a^	TATTGCTGGTGATGATGCTGCGG	23	39
	lst-miR-n37-2-5p^a^	TATTGCTGGTGATGATGCTGCGG	23	39
	lst-miR-n47-5p	TATTGCTGGTGATGATGCTGC	21	3
**miR-n84**	lst-miR-n86-1-5p^a^	TGGAGCGGGGCTGGGCTTT	19	5
	lst-miR-n86-2-5p^a^	TGGAGCGGGGCTGGGCTTT	19	5
**miR-n104**	lst-miR-n106-5p^a^	TGTTGTTGTTCTTGTTGGTGGGA	23	4
	lst-miR-n226-3p^a^	TGTTGTTGTTCTTGTTGGTGGGA	23	4
**miR-n136**	lst-miR-n138-1-3p^a^	TGCATCCGGCCAATTGACTG	20	4
	lst-miR-n138-2-3p^a^	TGCATCCGGCCAATTGACTG	20	4
**miR-n147**	lst-miR-n149-3p	TGGTAGCTACGCTTCGCCCT	20	3
	lst-miR-n187-3p	TGGTAGCTGCTGTGGTGGTGGT	22	6
**miR-n148**	lst-miR-n150-3p	CCAAGTGCACCCACGCGCCT	20	4
	lst-miR-n176-3p	TCAAGTGCAATGTGGTCGGTCC	22	3
**miR-n166**	lst-miR-n168-3p	TGTGGCTGCTGGGGGGGCTG	20	3
	lst-miR-n172-3p	TGTGGCTGCTGGGGGGGCTGCTG	23	19

a Ten miRNAs (lst-miR-n2, lst-miR-n3, lst-miR-n13, lst-miR-n17, lst-miR-n28, lst-miR-n29, lst-miR-n37, lst-miR-n84, lst-miR-n104, lst-miR-n136) have identical mature sequences, but were derived from different precursors.

Similar to the conserved miRNAs, the 274 novel miRNAs could also be divided into three types: (1) 12 miRNAs that represent both the 5′ and 3′ mature miRNAs of the corresponding six precursors, (2) 138 miRNAs that represent only the 5′ mature miRNAs and (3) 124 that represent only on the 3′ mature miRNAs (Table S2). However, the abundance of novel miRNAs was extremely low: of 501 total miRNAs, the novel miRNAs represented only 3.6% of the reads. The most highly expressed novel miRNA (lst-miR-n128-5p) had 16,450 reads; together with lst-miR-n114-5p (4308 reads) and lst-miR-n210-5p (1603 reads), these three miRNAs accounted for approximately 85% of all novel miRNA reads. The majority of novel miRNAs (238/274) had fewer than 20 reads, and almost half of them had fewer than 5 reads (150/274) (Table S2). All miRNAs identified here were aligned to the mitochondrial genome of *L. striatellus*, but no miRNA could be mapped, which suggests that these miRNAs were not produced from the mitochondrial genome, even although the mitochondrial genome can encode mitosRNAs [38].

### Validation of miRNAs

**Figure 5:**
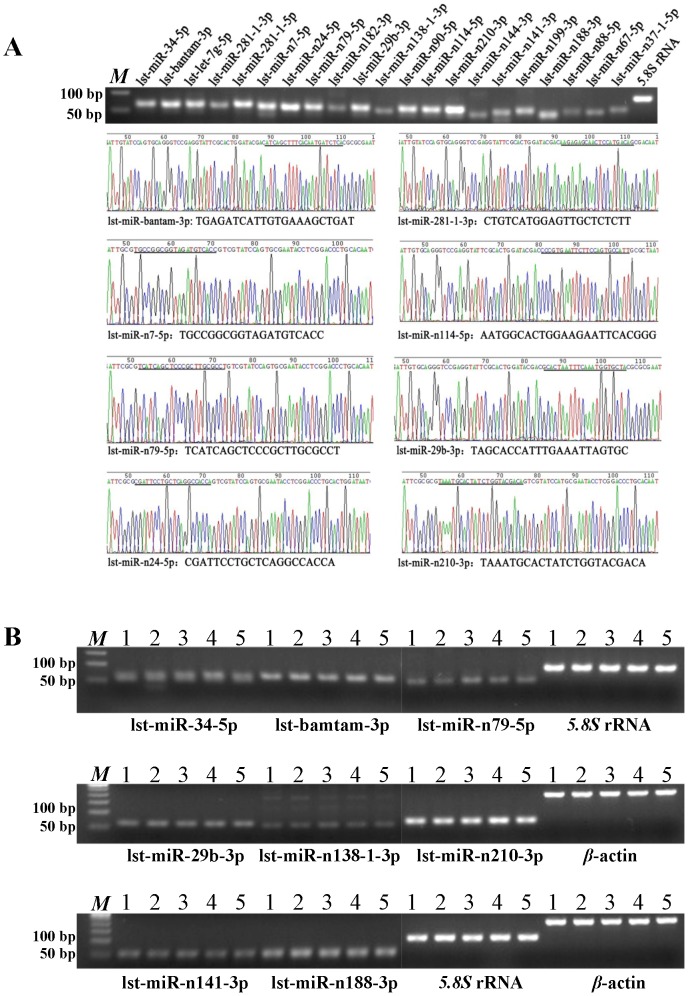
Expression validation of miRNAs by stem-loop RT-PCR. (A) 21 miRNAs, including 6 conserved and 15 novel miRNAs, were validated in mixed samples of *L. striatellus*; (B) validation of two conserved and five novel miRNAs in 1, second instar larvae; 2, fourth instar larvae; 3, female adults; 4, male adults and 5, mixed samples. β-actin and 5.8S rRNA were used as internal controls. *M*, marker.

The highly sensitive method of stem-loop reverse-transcription (RT)-PCR has been successfully applied to validate the expression of novel miRNAs in different species [49]–[51]. We randomly selected 21 miRNAs and designed specific primers (6 conserved and 15 novel) to validate their expression in *L. striatellus*. Eight of the validated miRNAs were further confirmed by sequencing (Figure 5A), suggesting that most miRNAs in *L. striatellus* identified by NGS are valid, even those miRNAs with fewer than five reads. Furthermore, the expression level of most miRNAs based on stem-loop RT-PCR was found to be consistent with the NGS read number, although some (lst-miR-n90-5p, lst-miR79-5p, lst-miR-n24-5p) were more highly expressed according to stem-loop RT-PCR, but had fewer than 50 reads (Figure 5A). This may be attributed to sequencing or cloning bias resulting from different methods [42].

To assess the expression status in different developmental stages of *L. striatellus*, second instar larvae, fourth instar larvae, and female and male adults were sampled to detect the expression levels of two conserved and five novel miRNAs. The results showed that there were no obvious differences in expression levels in different developmental stages of *L. striatellus* (Figure 5B).

### Conservation of miRNAs

Previous studies have shown that miRNAs are conserved between prokaryotes and eukaryotes [26], [32], [47], [52], [53]. In order to determine the conservation of miRNAs within animal species, 38 putative miRNA families with predicted miRNA hairpin structures in *L. striatellus* were compared to known miRNAs in 84 animal species from the miRBase and published NGS data (Table S4). These animal species are from nine phyla, including 30 in Arthropoda, 37 in Chordata, 7 in Nematoda, 5 in Platyhelminthes, and 1 species each in the remaining five phyla [54].

**Figure 6:**
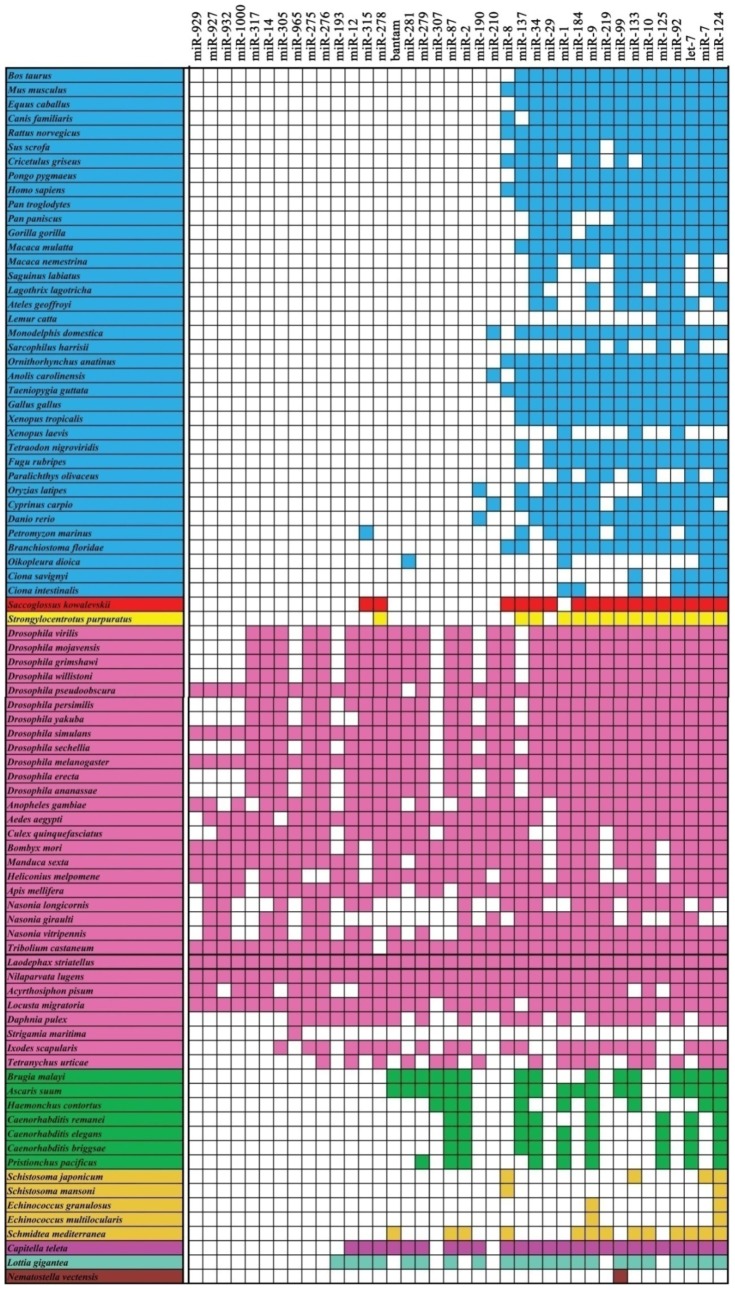
The conservation of miRNA families in 84 animal species. The miRNAs of 84 animal species belonging to nine phyla were extracted from miRBase and published NGS data, and the conservation was analyzed for 38 miRNA families with RNA secondary structure in *L. striatellus*. Colored boxes indicate the presence of the conserved miRNA family; the same color indicates similar species.

**Figure 7:**
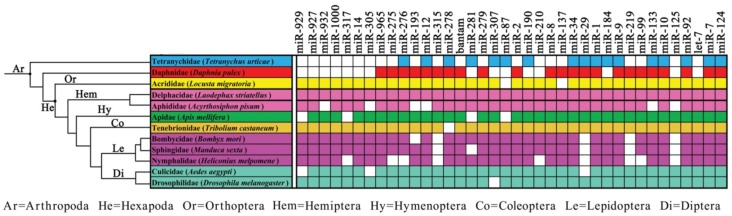
The conservation of miRNA families in Arthropoda. The Arthropoda phylum was divided into eight orders with different colors and 12 families; and one representative species was shown in each family. The phylogenetic topology tree was adapted from Wheat et al. (2013)[54].

All 38 conserved miRNA families were quite conserved in Arthropoda (Figure 6). *Nemateostella vetensis* (in the Cnidaria phylum) contained only one miRNA family (miR-99) that was conserved with all of the other phyla, suggesting that the Cnidaria phylum is distantly related to the others [55]. In the remaining seven phyla, 36–63% of miRNA families were conserved within the Arthropoda phylum. We further analyzed the conservation in 31 species of Arthropoda, which could be divided into 12 families, because only a few miRNA families of various phyla have been investigated. Not surprisingly, all 38 miRNA families were present in *N. lugens* [25], which is part of the same *Delphacidea* family as *L. striatellus*. This indicated that the miRNA families are quite conserved in *Delphacidea*. miRNA families were determined to be very conserved in Hexapoda, even though there were up to nine miRNA families that were absent in each group, and only four miRNA families (miR-315, miR-219, miR-125, and miR-29) were absent at the same time in three families of the Lepidoptera order (Figure 7). The *Aphididae* family (*Acyrthosiphon pisum*), the family most closely related to *Delphacidea*, had more miRNA families that were not detected than all of the other miRNA families. This may have been due to different sample collection methods and sequencing depth, as well as the delicate RNA processing procedure [34].

Many miRNAs families such as miR-10, let-7, miR-99, miR-125 are highly conserved among animal species. This implies that these conserved miRNAs have very important and similar functions. One of the oldest miRNAs in the animal species, the miR-99 family, is present in almost all animal species and is evolutionarily prehistoric [56]. Both let-7 and miR-125 families are derived from the miR-99. The let-7 family is conserved in a wide variety species of Deuterostomes and Protostomes, whereas the miR-125 family is absent in Annelida (*Lottia gigante*) [57]. Another ancient miRNA family, miR-10, has not been detected in the phylum Cnidaria or Nematoda, which may be due to the different sample collection methods and sequencing depth, as well as our delicate RNA processing procedure [34].

### Target prediction and function analysis of miRNAs

miRNAs induce cleavage of their target genes or inhibit their translation by interacting with them at specific sites [2], [6]. The high degree of complementarity between miRNAs and their targets facilitates the identification of the target genes by computational methods in plants [58]. However, it is difficult to identify miRNA targets in animals, because miRNAs bind to the 3′-UTR of their target genes with less than perfect complementarity [40], [59]. The miRanda software was applied to predict the putative target genes of all 501 miRNAs identified in *L. striatellus* [60]. A total of 2701 miRNA-target pairs were obtained from the *D. melanogaster* genome data for 367 miRNAs, which included 1032 for 150 conserved miRNAs and 2328 for 217 novel miRNAs (Table S5), with an average of 6.88 and 10.73 targets per miRNA, respectively. Six hundred and sixty-one genes were targeted by both conserved and novel miRNAs. Novel miRNAs appeared to have more targets than conserved miRNAs in *L. striatellus*. Of all of the 367 miRNAs, the novel miRNA lst-miR-n13-1-5p or lst-miR-n13-2-5p (their sequences are identical, but they are derived from different precursors) had the most potential targets (287), and lst-miR-1587 had the most potential targets (117) in the conserved miRNAs (Table S5). Of all of the targets, the gene FBgn0031077 had the highest number of potential miRNA regulators (30) (Table S6).

**Figure 8:**
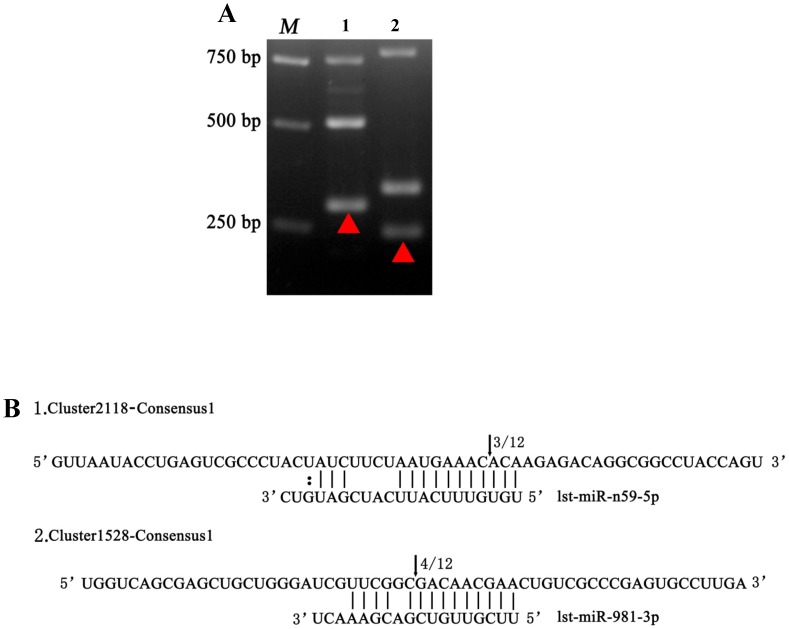
Verification of miRNA-mediated mRNA cleavage by RLM-5′ RACE. (A) Gel electrophoresis of 5′ RACE PCR products of target genes for miRNAs. The red triangles indicate the fragments of 1, Cluster2118-Consensus1 and 2, Cluster1528-Consensus1 cleaved by lst-miR-n59-5p and lst-miR-981-3p, respectively. (B) The upper sequences show the target mRNA, whereas the bottom sequences indicate its corresponding miRNA. Watson-Crick pairings are indicated by short black lines; the G:U wobble pair is indicated by a colon. Arrows indicate the cleavage sites with the frequency of cleavage.

Modified RNA ligase-mediated rapid amplification of cDNA ends (RLM-5′ RACE) was performed to assess the miRNA-guided target cleavage in *L. striatellus*. The miRNA targets Cluster2118-Consensus1 and Cluster1528-Consensus1 were cleaved by lst-miR-n59-5p and lst-miR-981-3p, respectively (Figure 8A). Unlike in plants [61]–[63], the cleavage frequency in *L. striatellus* was low (3/12 and 4/12) (Figure 8B), which may be because most animal miRNAs are an imperfect complement to their target genes and generally are considered to promote translational repression rather than cleavage. Although they have the function for cleaving target genes in animals, the mechanism of miRNA target cleavage with imperfect complementarity is still unclear [32], [64], [65].

**Figure 9:**
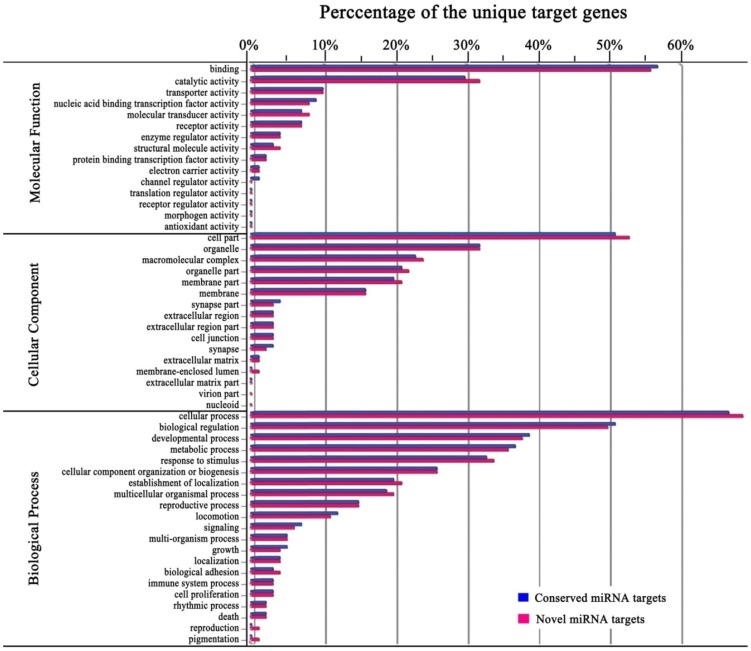
GO classification of the *L. striatellus* target genes. A total of 2103 putative target genes including 284 for conserved miRNAs and 1342 for novel miRNAs were assigned to the first-level of GO annotations of Biological Process, Cellular Component, and Molecular Function; and the potential target genes of conserved and novel miRNAs were classified into 50 and 52 functional groups at the second level of GO assignment, respectively.

To understand the biological function of miRNA in *L. striatellus*, all putative target genes were subjected to gene ontology (GO) functional classification by aligning them against fruit fly 3′-UTR databases (ftp://flybase.org/genomes/dmel/dmel_r5.55_FB2014_01/fasta/dmel-all-three_prime_UTR-r5.55.fasta.gz). Of the 2701 putative target genes, 2103 (78%) were assigned to the first-level of GO annotations of Biological Process, Cellular Component, and Molecular Function, which covered 284 target genes for conserved miRNAs and 1342 target genes for novel miRNAs. Furthermore, 531 target genes were co-regulated by novel and conserved miRNAs. At the second level of GO assignment, the potential target genes of conserved and novel miRNAs were classified into 50 and 52 functional groups, respectively (Figure 9).

The GO terms identified by our analysis suggested that the functions of the genes targeted by conserved and novel miRNAs are similar. The GO functional terms of ‘binding’ (in Molecular Function), ‘cell part’ (in Cellular Component), and ‘cellular process’ and ‘biological regulation’ (in Biological Process) accounted for more than 40% of all targeted genes analyzed (Figure 9). In fact, more than 95% of the miRNA targets were assigned to fewer than half of the GO terms in Molecular Function (6 and 6 of 15) and Cellular Component (6 and 5 of 16). Furthermore, terms of ‘virion part’ and ‘nucleoid’ in the Cellular Component annotation were not represented among conserved miRNA targets. The annotations of unique target genes of conserved and novel miRNAs compared to the GO databases provide helpful information for understanding the gene function and specific processes that have occurred throughout the evolution of *L. striatellus* (Figure 9).

## Conclusions

In this study, we used NGS to identify 501 mature miRNAs, which include 227 conserved and 274 novel miRNAs in the small brown planthopper (*L. striatellus*). Among these miRNAs, 69 conserved and 274 novel miRNAs were derived from 321 precursors. Stem-loop RT-PCR was applied to validate the miRNAs. Furthermore, 2701 unique target genes were predicted for 378 miRNAs, and the function of some miRNAs in the cleavage of target genes was confirmed by RLM-5′ RACE. This research will enrich the miRNA database of not only animals, but especially that of insects. Furthermore, this information provides the foundation for deciphering the relationship between miRNAs and their targets in *L. striatellus*.

## Materials and Methods

### Sample collection and total RNA extraction

Small brown planthoppers (*L. striatellus*) were fed in a climate chamber under a 12-hour light/12-hour dark cycle at 28°C at the Institute of Virology and Biotechnology, Zhejiang Academy of Agricultural Sciences, Hangzhou, China. The samples were collected from a mixed population composed of the first to fifth instar female and male nymphae and female and male adults. Total RNA was extracted using TRIzol reagent (Invitrogen, Carlsbad, CA, USA) according to the manufacturer's protocol. The extracted RNA was then tested and quantified by Nanodrop 2000.

### sRNA library construction and high-throughput sequencing

The sRNA library was constructed following the standard Solexa sRNA library protocol. First, the total RNA was run on 15% PAGE, and 15- to 30-nt sRNAs were recovered. Then, 5′ and 3′ RNA adaptors were ligated to sRNAs followed by reverse transcription into cDNAs. Finally, cDNAs were amplified by PCR and subjected to Solexa sequencing after purification.

### sRNA sequencing analysis and annotation

To obtain clean and unique sRNA reads, the raw data was filtered using several steps: (1) the incorrectly indexed reads were trimmed from the raw database; (2) the low-quality reads were removed; (3) the reads without 3′ adaptors were filtered; (4) sequences longer than 30 nt and shorter than 15 nt were clipped from the sRNA database; (5) finally, the reads of simple-repeat sequences were discarded. A large number of clean sRNA reads was obtained after all of these steps. The unique reads were acquired by removing the sRNAs that had two or more than two reads in the clean reads database.

All clean and unique reads were compared with the Drosophila genome using the Bowtie software. The mapped sequences were further divided into nine categories in accordance with their arrangement priority from exon sense, exon antisense, intron sense, intron antisense, rRNA, tRNA, miRNA, snRNA, and unannotated in the Rfam and GenBank databases. All the categories eliminate rRNA, tRNA, and snRNAs were used to precast mature miRNAs. All raw data have been submitted to the NCBI Short Read Archive under accession number SRP033399.

### Identification of conserved and novel miRNAs

In order to obtain the conserved miRNAs, the sRNAs (exon sense, exon antisense, intron sense, intron antisense, miRNA, unannotated sRNAs, and unmapped sRNAs) were aligned with the known miRNAs and miRNA precursors that were deposited in the miRBase 20.0 (http://www.mirbase.org/) and miRNAs from *N. lugens*, allowing no mismatches in the seed regions and no mismatches or one mismatch in the other sites. The sequences that matched known miRNAs were considered to be conserved miRNAs, and all of them were then mapped to the Drosophila genome, *N. lugens* transcriptome, and the mitochondrial genome of *L. striatellus* to obtain the potential precursors of the miRNAs. The remaining sequences were used to predict potential precursors of novel miRNAs against the Drosophila genome, *N. lugens* transcriptome, and the mitochondrial genome of *L. striatellus* as follows. The sequences 20 nt upstream and 100 nt downstream of the miRNA and the sequences 100 nt upstream and 20 nt downstream of the miRNA were extracted to predict the RNA secondary structure, and folded stem-loop structures were detected by Rfold (http://www.tbi.univie.ac.at/~ivo/RNA/RNAfold.html) and analyzed by Mireap (http://sourceforge.net/projects/mireap/) under the default settings. There are three characteristics in the structure of each typical miRNA precursor: (1) miRNA is located in one arm of the hairpin structure; (2) the RNA secondary structure of the precursor folds with a minimum energy less than −20 kcal/mol; and (3) the hairpin structure is located in an intron or intergenic region. The precursors of sRNAs that satisfied all criteria were considered to be miRNA candidates. The 5′ and 3′ notations were used to distinguish the miRNAs located in the 5′ end or 3′ end, respectively, of the corresponding hairpin precursor.

### Stem-loop RT-PCR and RLM-5′ RACE

Stem-loop RT-PCR was used to validate conserved and novel miRNAs. Total RNA was extracted from second instar larvae, fourth instar larvae, and female and male adults. *L. striatellus* cDNA was synthesized from respective total RNA with specific stem-loop primers designed according to previous work [49]. All stem-loop RT-PCR primers and miRNA-specific PCR primers are listed in Table S7. GoScript Reverse Transcriptase was used to process reverse transcriptase reactions that contained 1.5 µg of total RNA and 10 µM stem-loop RT-primer. The 20- µl reactions were incubated in a Mastercycler Nexus Gradient Thermal Cycler (Eppendorf, GER) at 25°C for 5 min, 42°C for 1 h, 70°C for 15 min, and then stored at 4°C for subsequent processing. The cDNAs were diluted 1∶10 to perform PCR for validation. The PCR mixture included 2 µl cDNA, 0.8 mM forward and reverse primers, 10× PCR buffer, 2.5 mM each of dNTPs, and 5U Tag polymerase (*TaKaRa Ex Taq*). The 20- µl reactions were incubated in a Mastercycler Nexus Gradient Thermal Cycler in a 96-well plate at 95°C for 5 min, followed by 35 cycles of 95°C for 15 s, 62°C for 30 s, and 30 s at 72°C, and then stored at 4°C for subsequent processing. The PCR products were separated by electrophoresis with 3% agarose gel containing ethidium bromide and photographed under ultraviolet light.

RLM-5′ RACE was performed to confirm the cleavage of target genes for miRNA using a 5′ RACE kit (Takara) [61], [62]. An RNA oligo adapter was directly ligated to the total RNA extracted from the mixed *L. striatellus* specimens without calf intestinal phosphatase or tobacco acid pyrophosphatase treatment. The 10- µl reactions were incubated in a Mastercycler Nexus Gradient Thermal Cycler (Eppendorf, GER) at 30°C for 10 min, 42°C for 1 h, 70°C for 15 min, and then stored at 4°C for subsequent processing. The 5′ RACE outer primer, inner primer, and two paired gene-specific primers were used for nested PCR. The primers for the target genes were based on the transcript sequences of *L. striatellus* (unpublished data) (Table S8). The 50- µl outer PCR reactions were conducted in a Mastercycler Nexus Gradient Thermal Cycler at 94°C for 3 min, followed by 20 cycles of 94°C for 30 s, 55°C for 30 s, 1 min at 72°C, and then stored at 4°C for subsequent processing. The 50- µl inner PCR reactions were conducted at the same conditions, but they were reacted for 30 cycles according to the 5′ RACE instructions. The PCR products were then gel-purified and cloned, and at least 12 independent clones were sequenced to determine cleavage frequency.

### Target prediction and GO annotation of miRNAs

Putative target genes for all miRNAs were predicted by alignment to the 3′-UTR sequences of the Drosophila genome, which were downloaded from FlyBAse (http://flybase.org/). miRanda v3.1 was selected as the prediction tool, with the minimum free energy less than −25 kcal/mol [60]. Function annotation of the predicted target genes by GO terms was conducted using the GOslim tool in Blast2GO software (http://www.blast2go.org/).

## Supporting Information

Figure S1 **Length distribution of sRNAs that were mapped to the mitochondrial genome of** ***L. striatellus*** **.** All sRNA clean reads were mapped on the mitochondrial genome of *L. striatellus*, and unique reads and redundant reads were shown in blue and red, respectively.
(PPTX)

Figure S2 **RNA secondary structures of miRNA precursors in** ***L. striatellus*** **.** The folded stem-loop structures were detected by Rfold (http://www.tbi.univie.ac.at/~ivo/RNA/RNAfold.html) and analyzed by mireap (http://sourceforge.net/projects/mireap/) under the default settings. The precursors of sRNAs that fit all miRNA filter criteria were considered to be miRNA candidates.
(PPTX)

Table S1 **Conserved mature miRNAs and miRNA families identified in** ***L. striatellus*** **.**
(XLSX)

Table S2 **The property of miRNAs and their corresponding precursors in** ***L. striatellus*** **.**
(XLSX)

Table S3 **Mature miRNAs and miRNA families with hairpin structures in** ***L. striatellus*** **.**
(XLSX)

Table S4 **Summary of 85 animal species that were used for the conservation study of miRNAs.**
(XLSX)

Table S5 **The 367 miRNAs identified in** ***L. striatellus*** **and their potential target genes.**
(XLS)

Table S6 **The 2701 potential target genes and their corresponding miRNAs in** ***L. striatellus*** **.**
(XLS)

Table S7 **The primer sequences of stem-loop RT.**
(DOCX)

Table S8 **The outer and inner primer sequences of RLM-5′RACE.**
(DOCX)
