# Supplementary figures and images for: Identification and Characterization of MicroRNAs in Small Brown Planthopper (Laodephax striatellus) by Next-Generation Sequencing
Source: PLoS One. 2014 Jul 24;9(7):e103041. doi: 10.1371/journal.pone.0103041 (PMC4109989; doi:10.1371/journal.pone.0103041)

## Slide 1
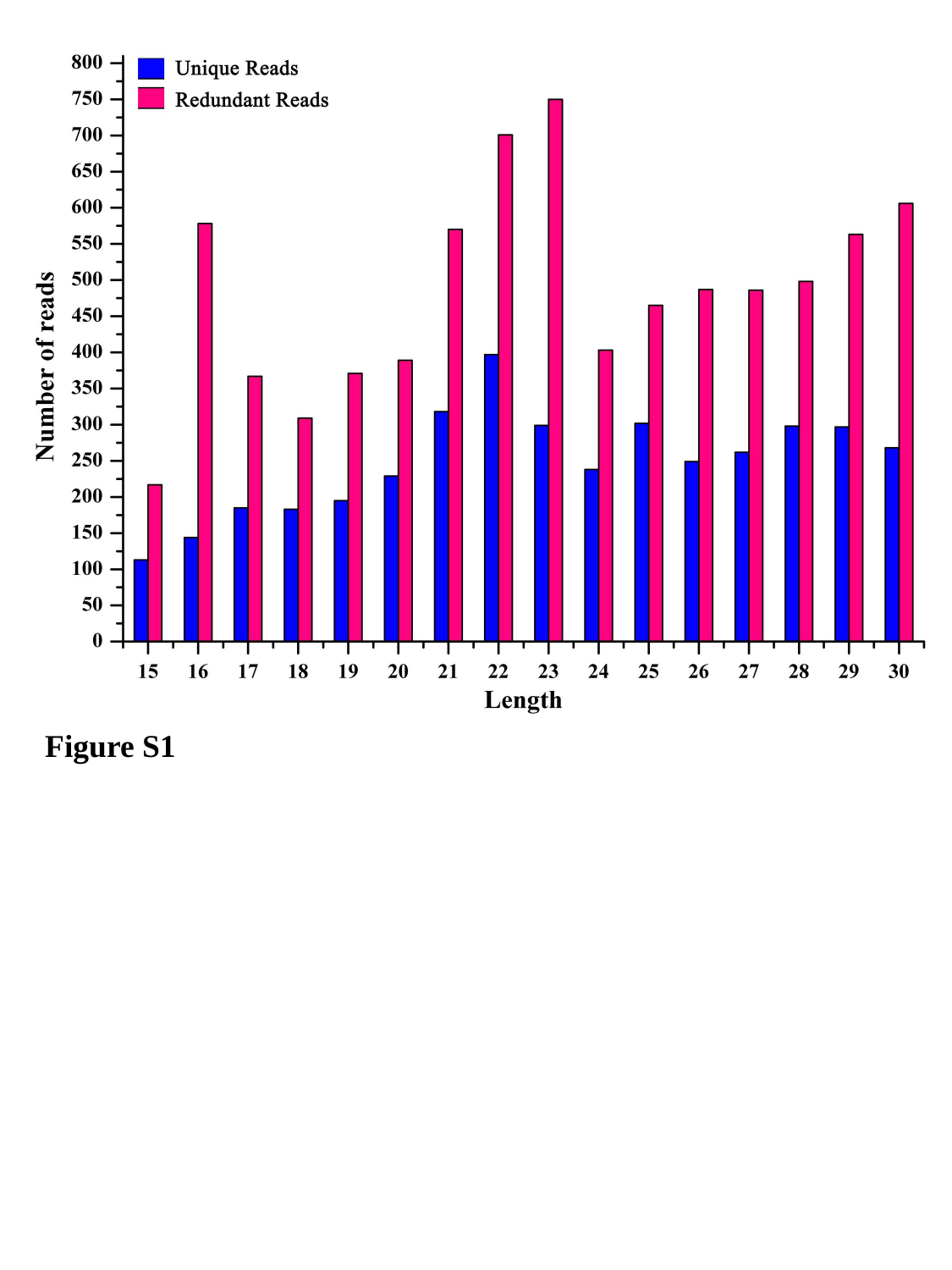

Figure S1

Supplement: Figure S1 — Length distribution of sRNAs that were mapped to the mitochondrial genome of L. striatellus . All sRNA clean reads were mapped on the mitochondrial genome of L. striatellus, and unique reads and redundant reads were shown in blue and red, respectively. (PPTX) [file pone.0103041.s001.pptx]
