# Supplementary material for: Identification and Characterization of MicroRNAs in Small Brown Planthopper (Laodephax striatellus) by Next-Generation Sequencing
Source: PLoS One. 2014 Jul 24;9(7):e103041. doi: 10.1371/journal.pone.0103041 (PMC4109989; doi:10.1371/journal.pone.0103041)
Supplement: Figure S2 — RNA secondary structures of miRNA precursors in L. striatellus . The folded stem-loop structures were detected by Rfold (http://www.tbi.univie.ac.at/~ivo/RNA/RNAfold.html) and analyzed by mireap (http://sourceforge.net/projects/mireap/) under the default settings. The precursors of sRNAs that fit all miRNA filter criteria were considered to be miRNA candidates. (PPTX) [file pone.0103041.s002.pptx]

## Slide 1
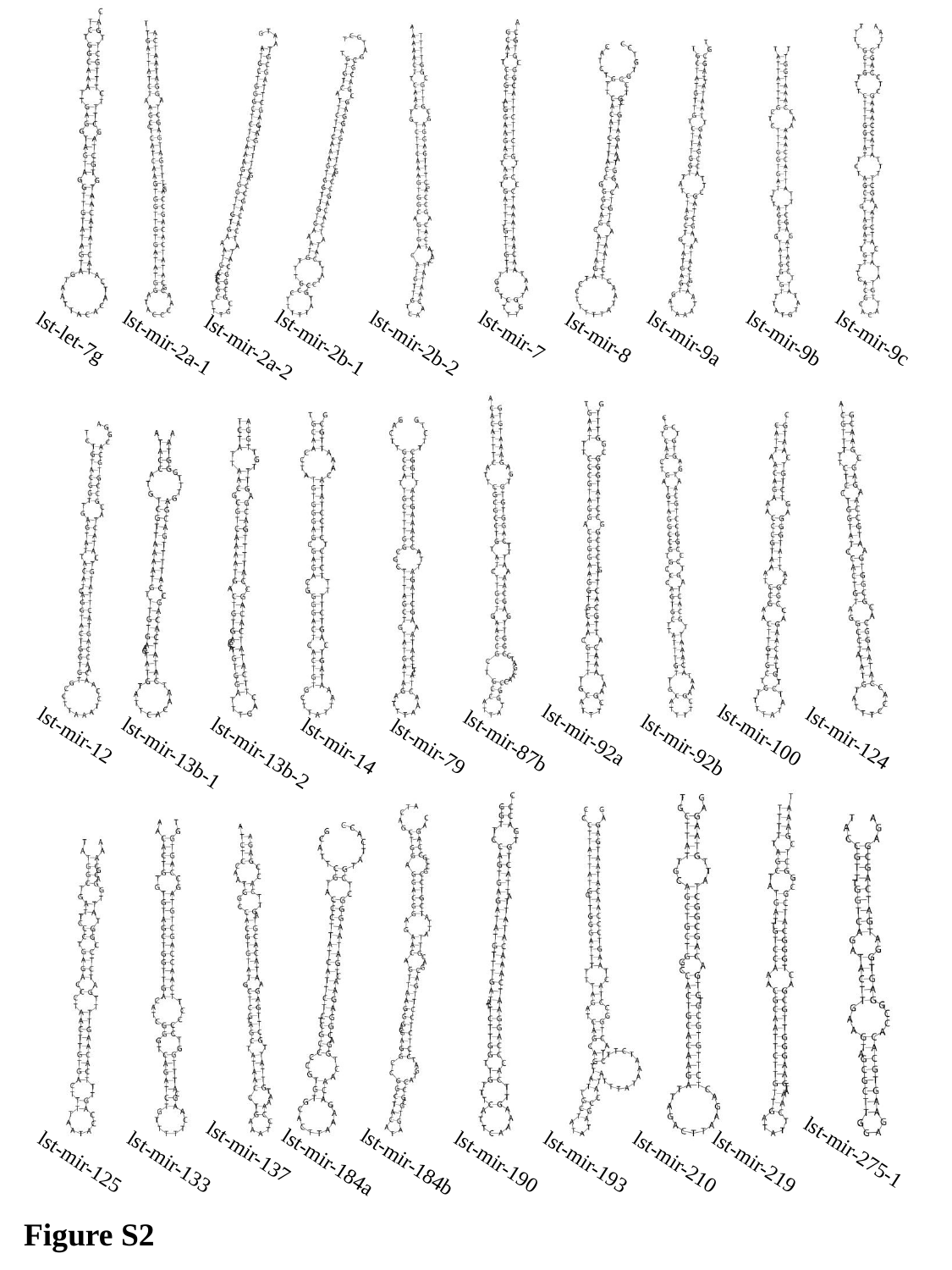

lst-mir-7
lst-let-7g
lst-mir-8
lst-mir-9a
lst-mir-9c
lst-mir-9b
lst-mir-2b-1
lst-mir-2a-1
lst-mir-2b-2
lst-mir-2a-2
lst-mir-12
lst-mir-92a
lst-mir-100
lst-mir-124
lst-mir-87b
lst-mir-92b
lst-mir-14
lst-mir-79
lst-mir-13b-1
lst-mir-13b-2
lst-mir-137
lst-mir-275-1
lst-mir-125
lst-mir-133
lst-mir-184a
lst-mir-184b
lst-mir-190
lst-mir-193
lst-mir-210
lst-mir-219
Figure S2

## Slide 2
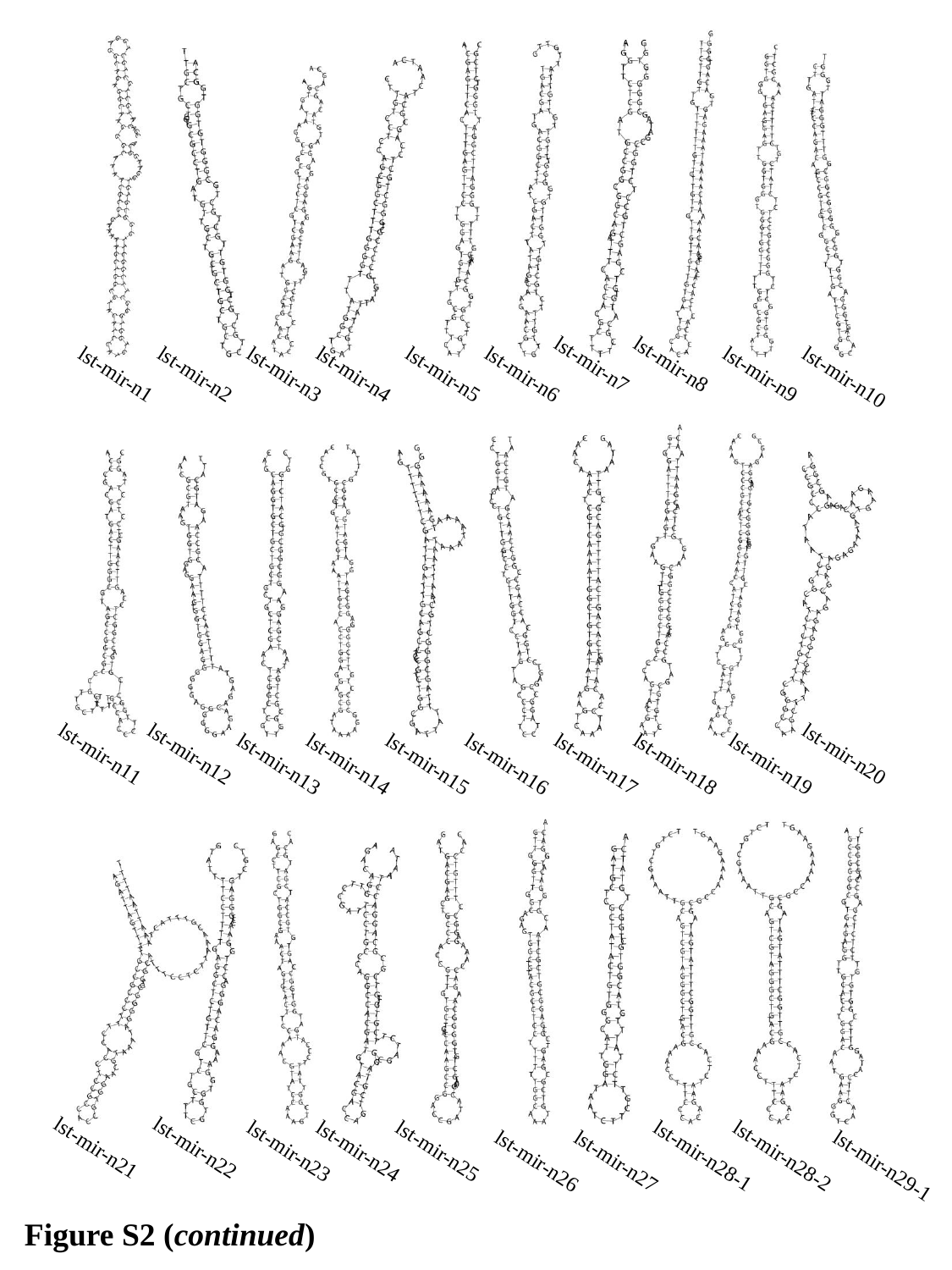

lst-mir-n7
lst-mir-n8
lst-mir-n2
lst-mir-n3
lst-mir-n4
lst-mir-n5
lst-mir-n6
lst-mir-n9
lst-mir-n1
lst-mir-n10
lst-mir-n11
lst-mir-n12
lst-mir-n20
lst-mir-n14
lst-mir-n13
lst-mir-n15
lst-mir-n16
lst-mir-n18
lst-mir-n17
lst-mir-n19
lst-mir-n22
lst-mir-n21
lst-mir-n23
lst-mir-n24
lst-mir-n25
lst-mir-n28-1
lst-mir-n28-2
lst-mir-n27
lst-mir-n26
lst-mir-n29-1
Figure S2 (continued)

## Slide 3
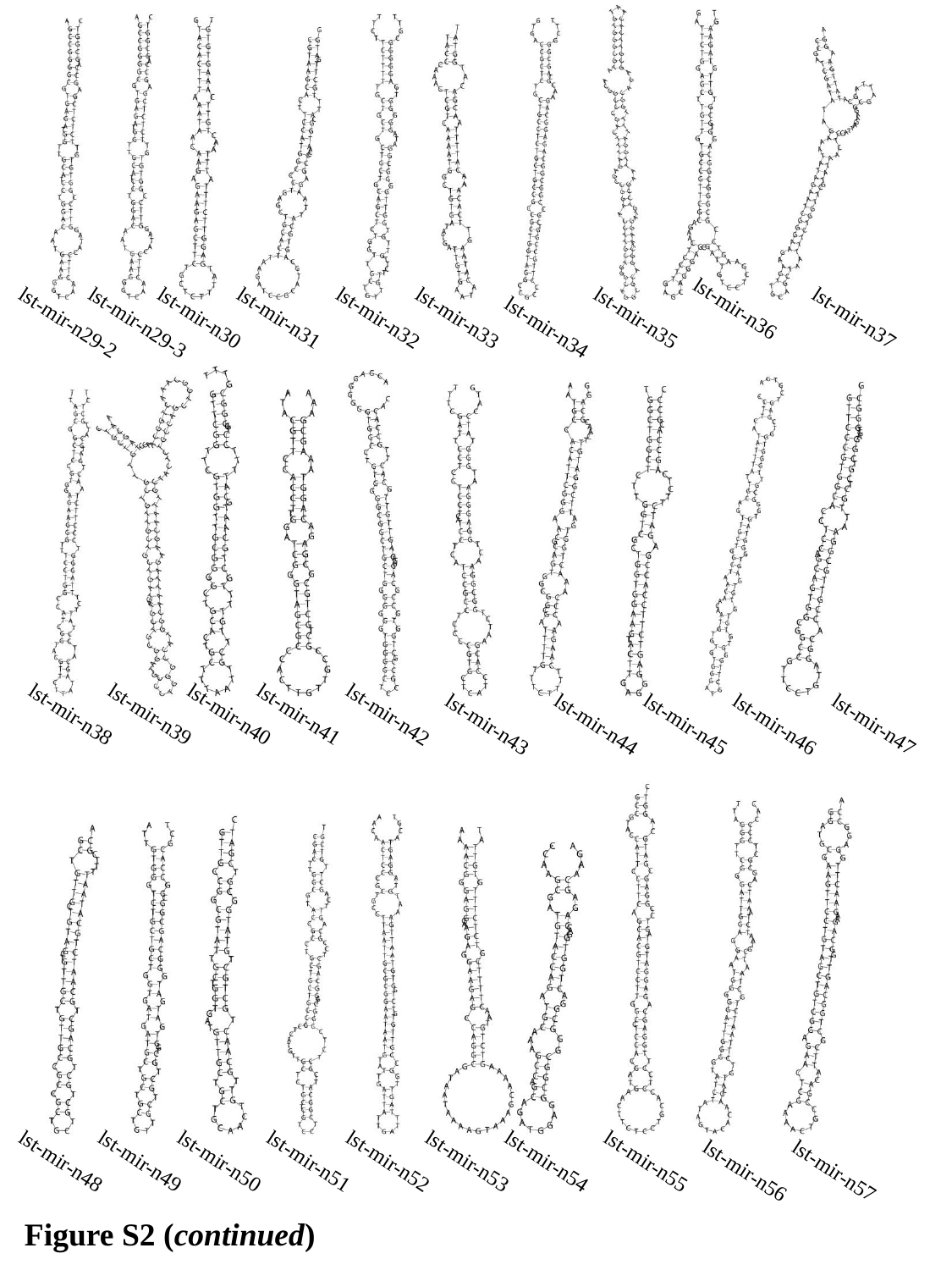

lst-mir-n36
lst-mir-n31
lst-mir-n30
lst-mir-n33
lst-mir-n35
lst-mir-n37
lst-mir-n32
lst-mir-n29-2
lst-mir-n29-3
lst-mir-n34
lst-mir-n38
lst-mir-n40
lst-mir-n41
lst-mir-n42
lst-mir-n39
lst-mir-n44
lst-mir-n43
lst-mir-n45
lst-mir-n46
lst-mir-n47
lst-mir-n51
lst-mir-n52
lst-mir-n53
lst-mir-n48
lst-mir-n49
lst-mir-n50
lst-mir-n54
lst-mir-n55
lst-mir-n56
lst-mir-n57
Figure S2 (continued)

## Slide 4
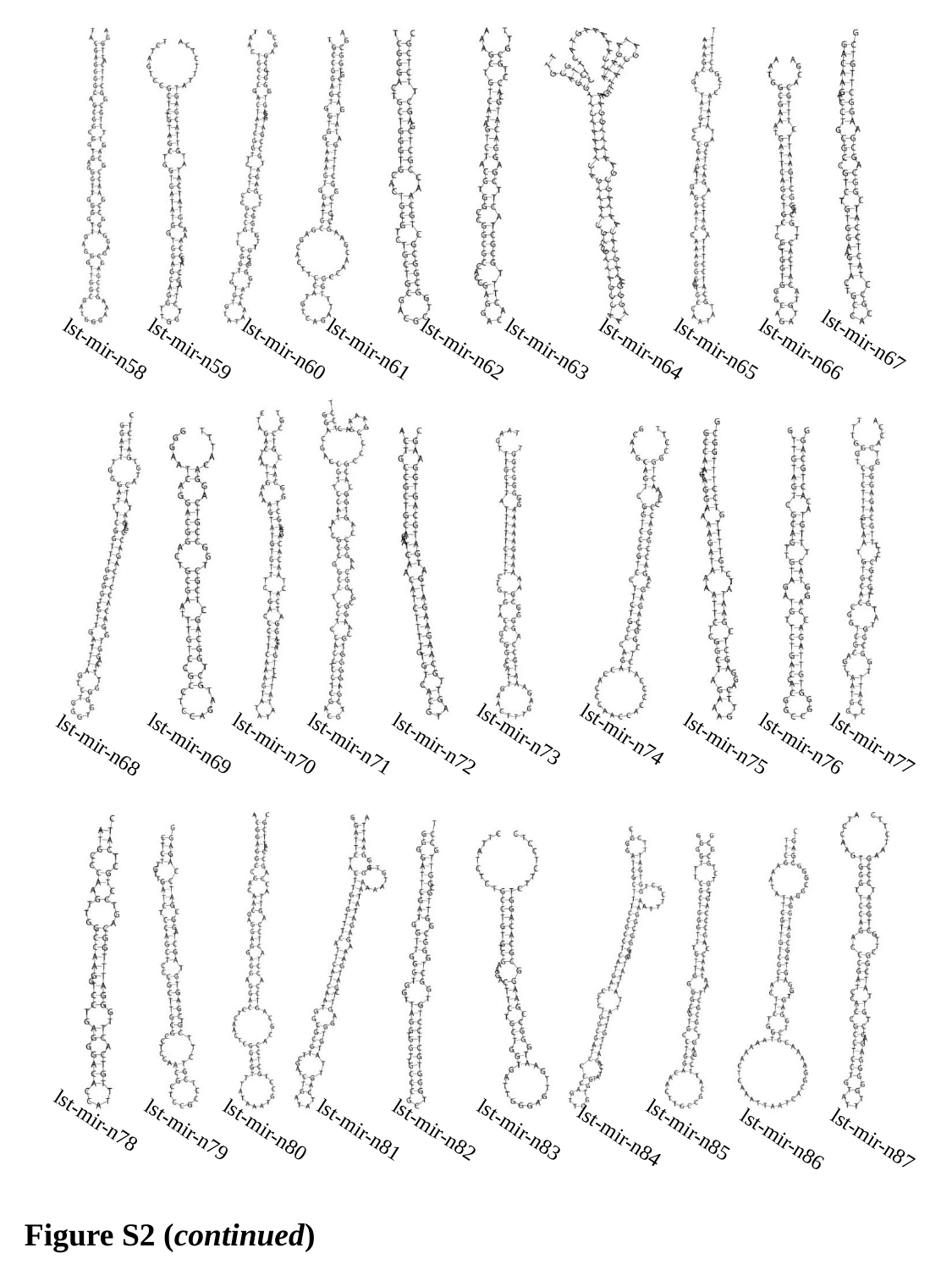

lst-mir-n67
lst-mir-n61
lst-mir-n65
lst-mir-n58
lst-mir-n59
lst-mir-n60
lst-mir-n62
lst-mir-n63
lst-mir-n66
lst-mir-n64
lst-mir-n74
lst-mir-n73
lst-mir-n76
lst-mir-n69
lst-mir-n70
lst-mir-n71
lst-mir-n77
lst-mir-n72
lst-mir-n75
lst-mir-n68
lst-mir-n78
lst-mir-n79
lst-mir-n81
lst-mir-n82
lst-mir-n83
lst-mir-n85
lst-mir-n80
lst-mir-n84
lst-mir-n86
lst-mir-n87
Figure S2 (continued)

## Slide 5
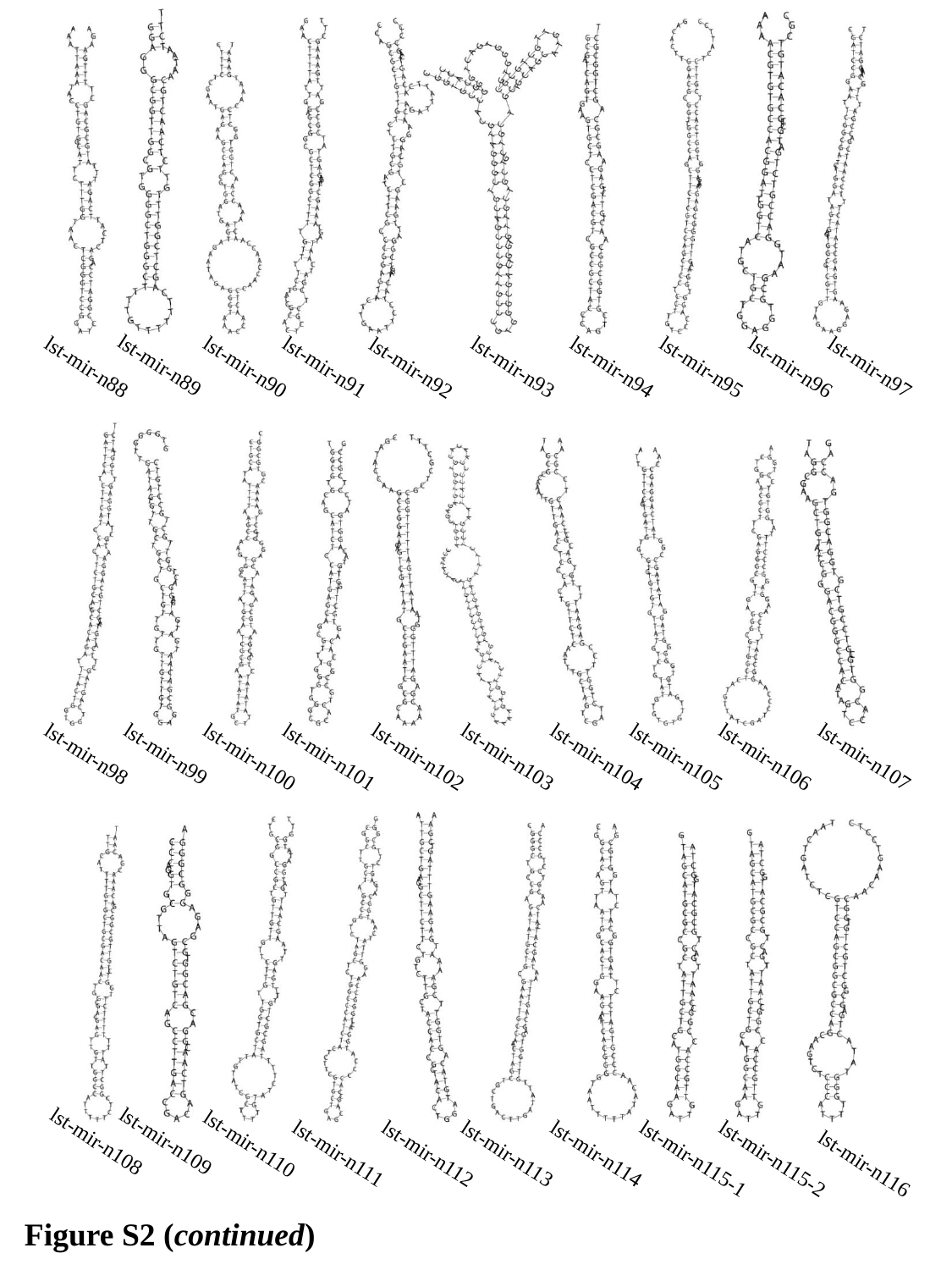

lst-mir-n89
lst-mir-n96
lst-mir-n88
lst-mir-n90
lst-mir-n91
lst-mir-n93
lst-mir-n95
lst-mir-n97
lst-mir-n94
lst-mir-n92
lst-mir-n98
lst-mir-n99
lst-mir-n105
lst-mir-n106
lst-mir-n100
lst-mir-n101
lst-mir-n102
lst-mir-n103
lst-mir-n104
lst-mir-n107
lst-mir-n108
lst-mir-n109
lst-mir-n110
lst-mir-n111
lst-mir-n112
lst-mir-n114
lst-mir-n113
lst-mir-n115-2
lst-mir-n115-1
lst-mir-n116
Figure S2 (continued)

## Slide 6
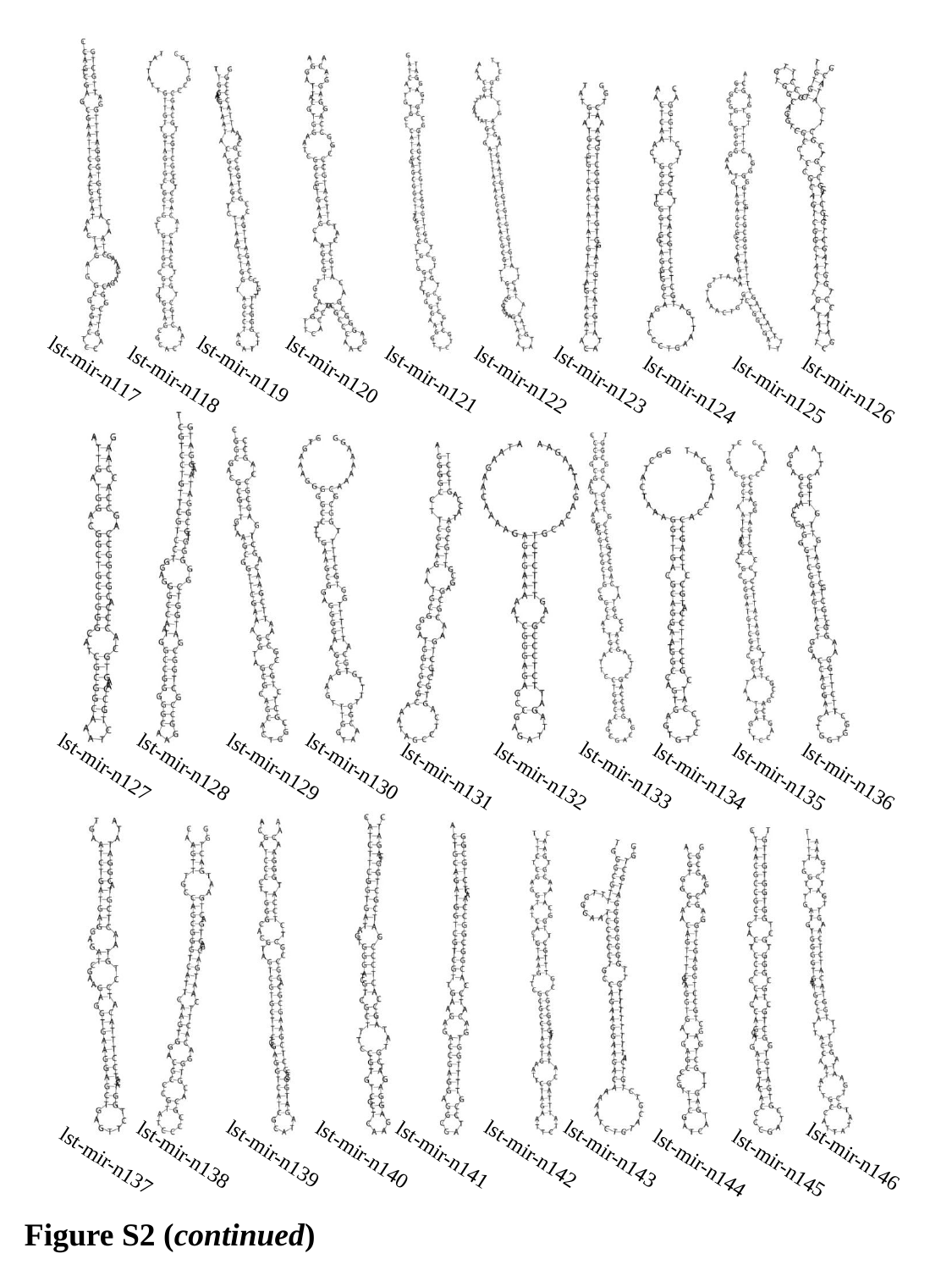

lst-mir-n117
lst-mir-n119
lst-mir-n120
lst-mir-n118
lst-mir-n121
lst-mir-n122
lst-mir-n123
lst-mir-n124
lst-mir-n126
lst-mir-n125
lst-mir-n127
lst-mir-n130
lst-mir-n128
lst-mir-n129
lst-mir-n133
lst-mir-n132
lst-mir-n131
lst-mir-n135
lst-mir-n134
lst-mir-n136
lst-mir-n139
lst-mir-n142
lst-mir-n143
lst-mir-n140
lst-mir-n141
lst-mir-n138
lst-mir-n146
lst-mir-n137
lst-mir-n145
lst-mir-n144
Figure S2 (continued)

## Slide 7
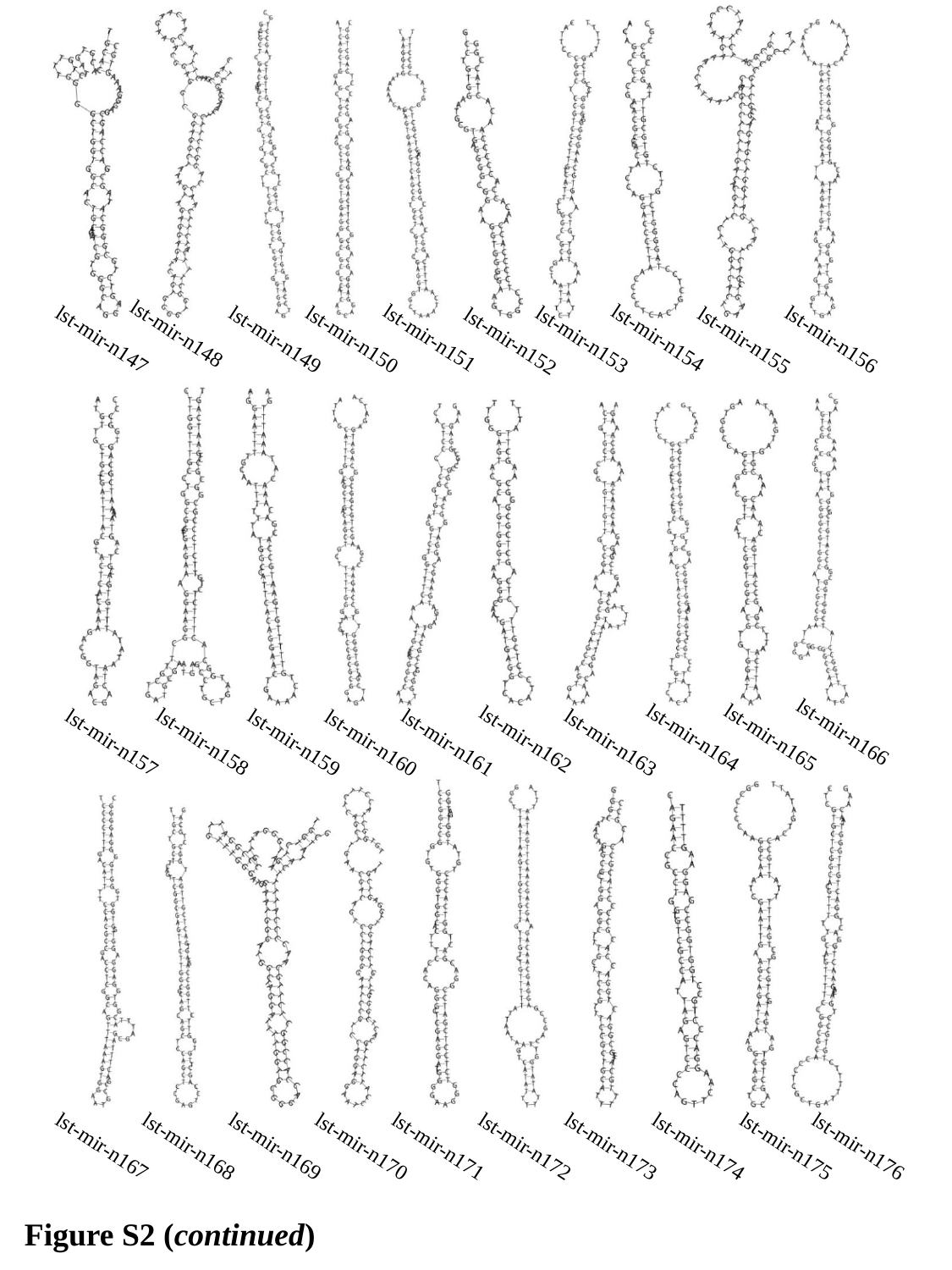

lst-mir-n148
lst-mir-n154
lst-mir-n151
lst-mir-n156
lst-mir-n147
lst-mir-n149
lst-mir-n150
lst-mir-n153
lst-mir-n155
lst-mir-n152
lst-mir-n166
lst-mir-n164
lst-mir-n165
lst-mir-n162
lst-mir-n158
lst-mir-n163
lst-mir-n157
lst-mir-n159
lst-mir-n161
lst-mir-n160
lst-mir-n167
lst-mir-n176
lst-mir-n170
lst-mir-n172
lst-mir-n168
lst-mir-n169
lst-mir-n171
lst-mir-n173
lst-mir-n175
lst-mir-n174
Figure S2 (continued)

## Slide 8
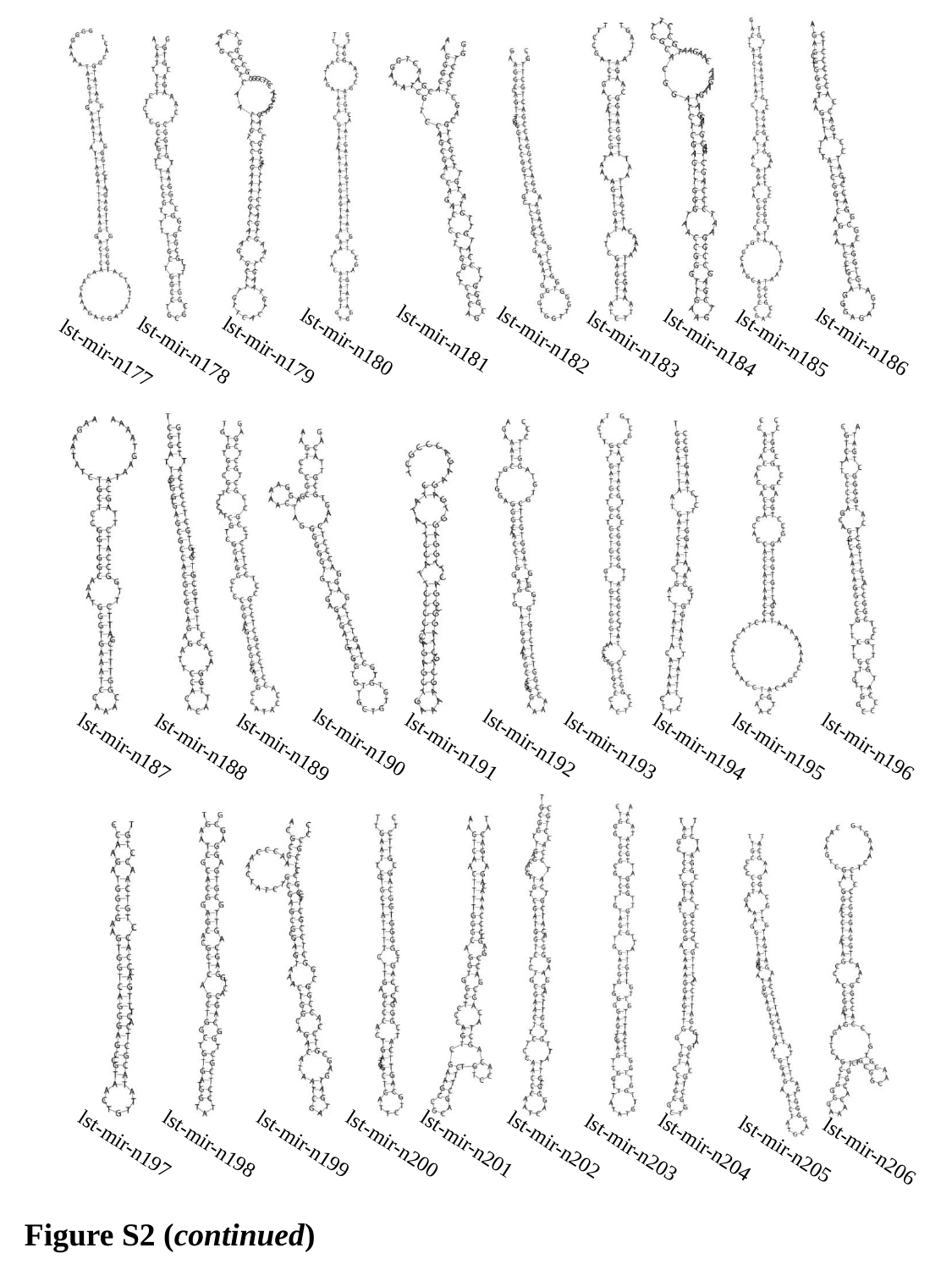

lst-mir-n181
lst-mir-n182
lst-mir-n180
lst-mir-n186
lst-mir-n184
lst-mir-n185
lst-mir-n183
lst-mir-n179
lst-mir-n178
lst-mir-n177
lst-mir-n190
lst-mir-n192
lst-mir-n193
lst-mir-n196
lst-mir-n187
lst-mir-n191
lst-mir-n195
lst-mir-n194
lst-mir-n188
lst-mir-n189
lst-mir-n198
lst-mir-n197
lst-mir-n199
lst-mir-n200
lst-mir-n201
lst-mir-n202
lst-mir-n204
lst-mir-n203
lst-mir-n205
lst-mir-n206
Figure S2 (continued)

## Slide 9
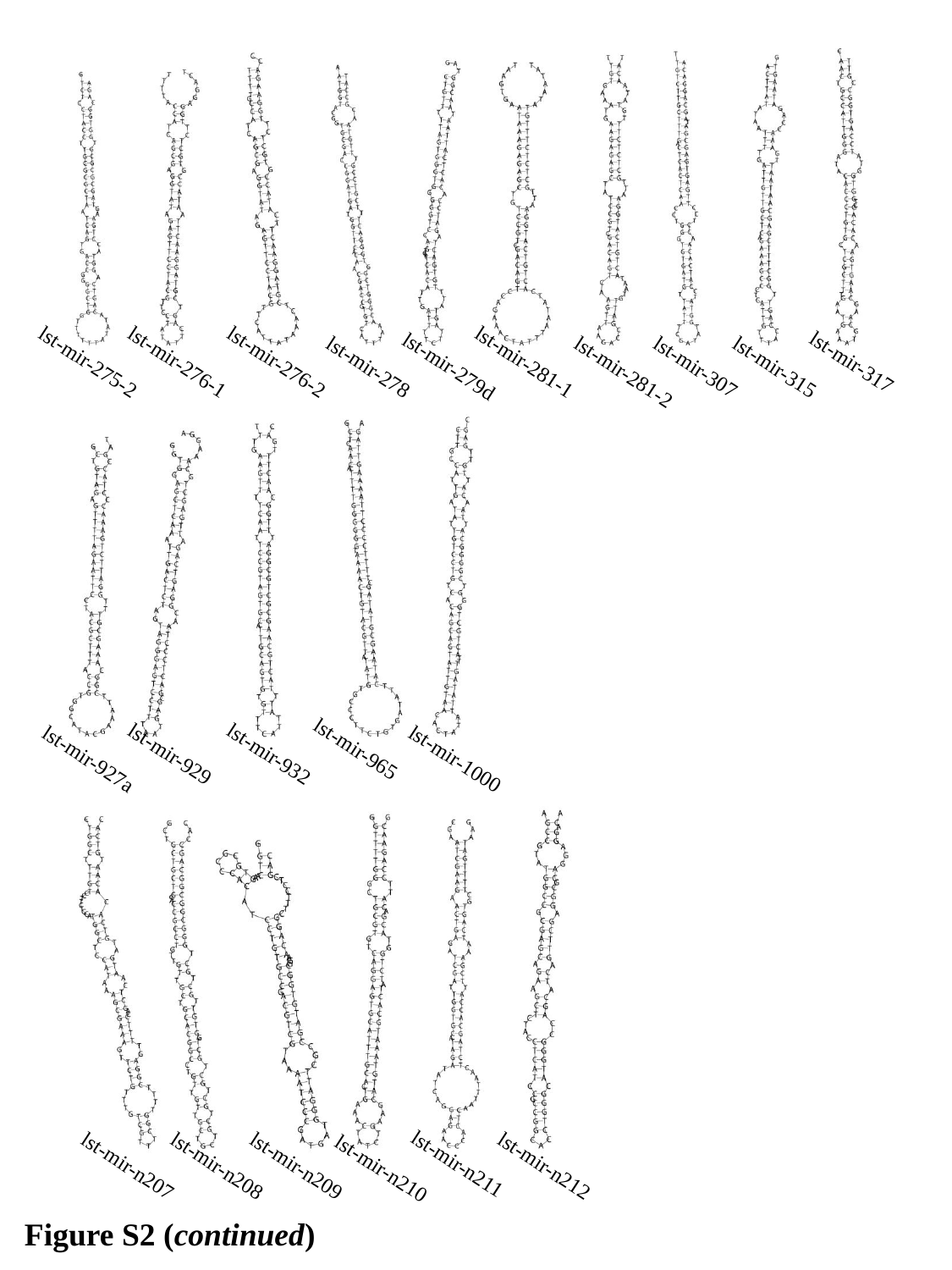

lst-mir-281-1
lst-mir-276-1
lst-mir-276-2
lst-mir-317
lst-mir-275-2
lst-mir-279d
lst-mir-278
lst-mir-307
lst-mir-315
lst-mir-281-2
lst-mir-965
lst-mir-929
lst-mir-932
lst-mir-1000
lst-mir-927a
lst-mir-n211
lst-mir-n212
lst-mir-n207
lst-mir-n208
lst-mir-n209
lst-mir-n210
Figure S2 (continued)

## Slide 10
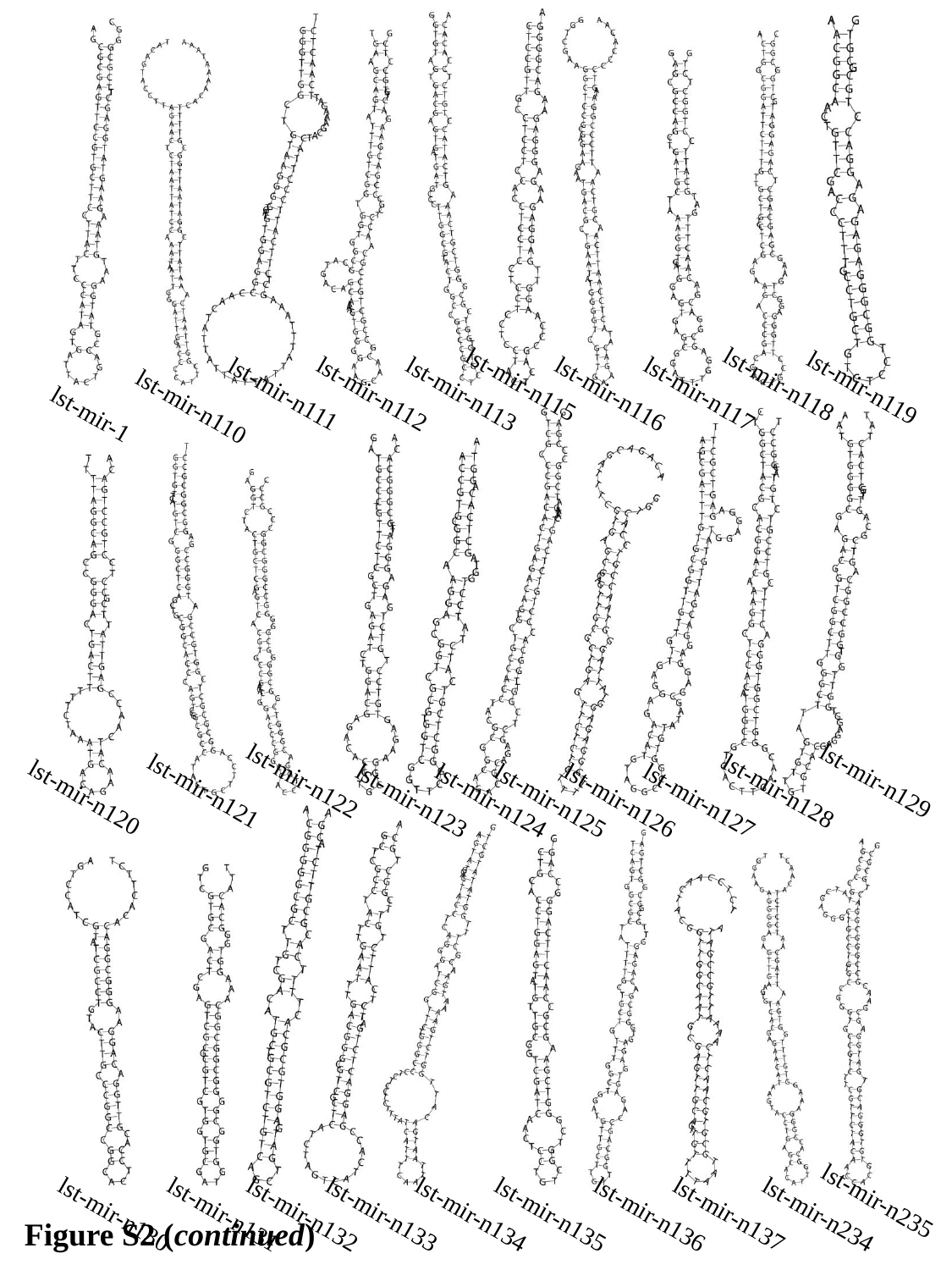

lst-mir-n115
lst-mir-n118
lst-mir-n119
lst-mir-n112
lst-mir-n117
lst-mir-n111
lst-mir-n113
lst-mir-n116
lst-mir-n110
lst-mir-1
lst-mir-n122
lst-mir-n129
lst-mir-n128
lst-mir-n121
lst-mir-n120
lst-mir-n127
lst-mir-n125
lst-mir-n126
lst-mir-n123
lst-mir-n124
lst-mir-n235
lst-mir-n130
lst-mir-n132
lst-mir-n136
lst-mir-n131
lst-mir-n133
lst-mir-n134
lst-mir-n135
lst-mir-n137
lst-mir-n234
Figure S2 (continued)

## Slide 11
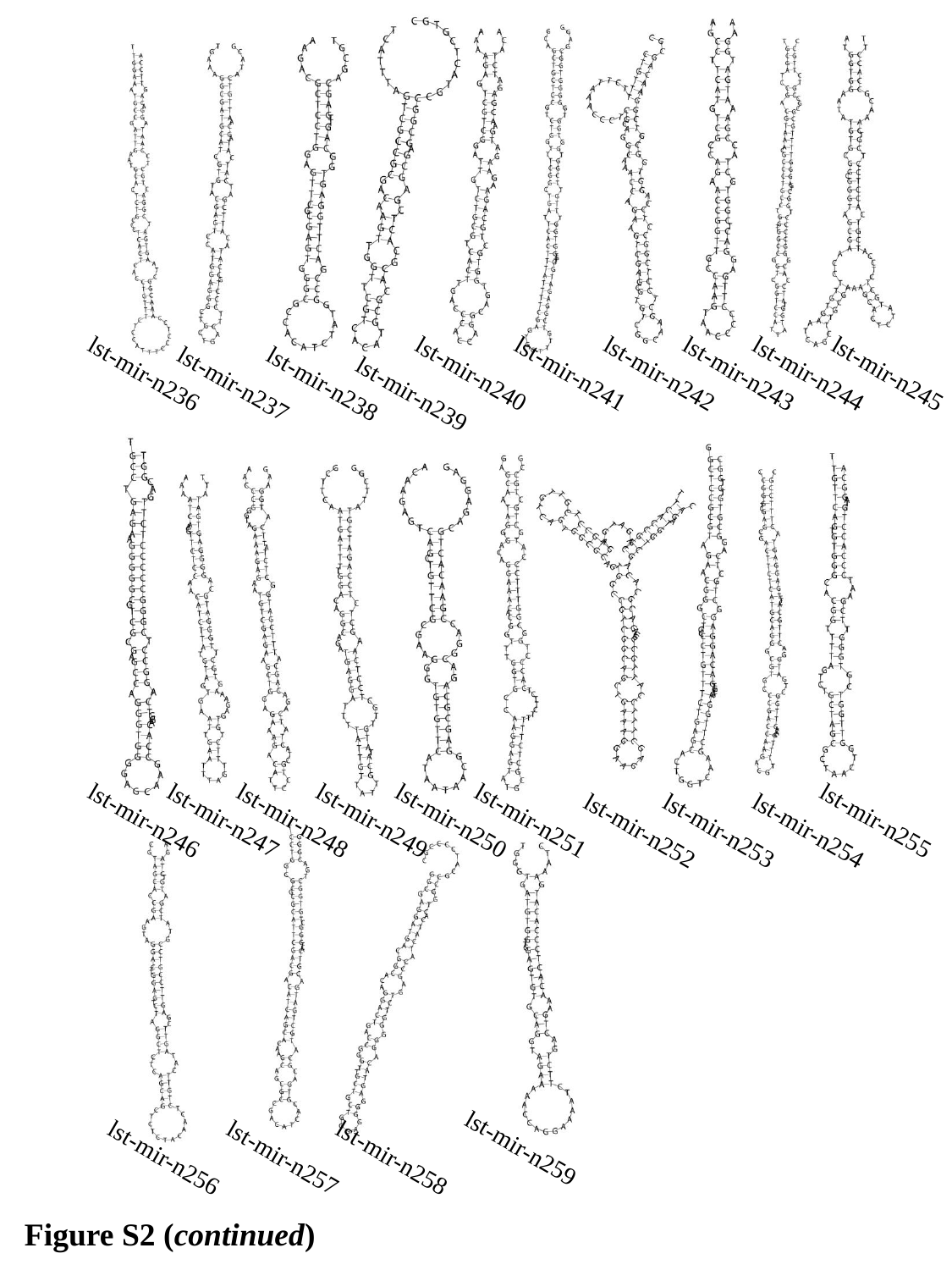

lst-mir-n236
lst-mir-n243
lst-mir-n244
lst-mir-n245
lst-mir-n240
lst-mir-n241
lst-mir-n242
lst-mir-n238
lst-mir-n237
lst-mir-n239
lst-mir-n249
lst-mir-n251
lst-mir-n246
lst-mir-n248
lst-mir-n250
lst-mir-n255
lst-mir-n247
lst-mir-n253
lst-mir-n254
lst-mir-n252
lst-mir-n259
lst-mir-n256
lst-mir-n257
lst-mir-n258
Figure S2 (continued)
