# Supplementary material for: Identification and Characterization of MicroRNAs in Small Brown Planthopper (Laodephax striatellus) by Next-Generation Sequencing
Source: PLoS One. 2014 Jul 24;9(7):e103041. doi: 10.1371/journal.pone.0103041 (PMC4109989; doi:10.1371/journal.pone.0103041)
Supplement: Table S7 — The primer sequences of stem-loop RT. (DOCX) [file pone.0103041.s009.docx]

**Table S7. The primer sequences of stem-loop RT**

| miRNA ID | Primers | Sequences |
| --- | --- | --- |
| lst-miR-34-5p |  | TGGCAGTGTGGTTAGCTGGTTGT |
|  | RT | GTCGTATCCAGTGCAGGGTCCGAGGTATTCGCACTGGATACGACACAACC |
|  | Forward | CGCGCGTGGCAGTGTGGT |
|  | Reverse | GTGCAGGGTCCGAGGT |
| lst-bantam-3p |  | TGAGATCATTGTGAAAGCTGAT |
|  | RT | GTCGTATCCAGTGCAGGGTCCGAGGTATTCGCACTGGATACGACATCAGC |
|  | Forward | CGCGCGTGAGATCATTGTG |
|  | Reverse | GTGCAGGGTCCGAGGT |
| lst-let-7g-5p |  | TGAGGTAGTAGGTTGTATAGT |
|  | RT | GTCGTATCCAGTGCAGGGTCCGAGGTATTCGCACTGGATACGACACTATA |
|  | Forward | CGCGCGTGAGGTAGTAGG |
|  | Reverse | GTGCAGGGTCCGAGGT |
| lst-miR-281-1-3p |  | CTGTCATGGAGTTGC**TCTCTT** |
|  | RT | GTCGTATCCAGTGCAGGGTCCGAGGTATTCGCACTGGATACGAC**AAGAGA** |
|  | Forward | CGTCGCTGTCATGGAGTTGC |
|  | Reverse | GTGCAGGGTCCGAGGT |
| lst-miR-281-1-5p |  | AAGAGAGCTATCCGTC**GACAGT** |
|  | RT | GTCGTATCCAGTGCAGGGTCCGAGGTATTCGCACTGGATACGAC**ACTGTC** |
|  | Forward | CGCGCGAAGAGAGCTATCCG |
|  | Reverse | GTGCAGGGTCCGAGGT |
| lst-miR-n7-5p |  | TGCCGGCGGTAGAT**GTCACC** |
|  | RT | GTCGTATCCAGTGCAGGGTCCGAGGTATTCGCACTGGATACGACGGTGAC |
|  | Forward | GCGTGCCGGCGGTAGAT |
|  | Reverse | GTGCAGGGTCCGAGGT |
| lst-miR-n24-5p |  | CGATTCCTGCTCAGG**CCACCA** |
|  | RT | GTCGTATCCAGTGCAGGGTCCGAGGTATTCGCACTGGATACGAC**TGGTGG** |
|  | Forward | CGCGCGATTCCTGCTCAG |
|  | Reverse | GTGCAGGGTCCGAGGT |
| lst-miR-n79-5p |  | TCATCAGCTCCCGCTT**GCGCCT** |
|  | RT | GTCGTATCCAGTGCAGGGTCCGAGGTATTCGCACTGGATACGAC**AGGCGC** |
|  | Forward | CGCGTCATCAGCTCCCGC |
|  | Reverse | GTGCAGGGTCCGAGGT |
| lst-miR-n182-3p |  | TCGGCGCAGAAGGAC**CGGACC** |
|  | RT | GTCGTATCCAGTGCAGGGTCCGAGGTATTCGCACTGGATACGAC**GGTCCG** |
|  | Forward | GCGTCGGCGCAGAAGGA |
|  | Reverse | GTGCAGGGTCCGAGGT |
| lst-miR-29b-3p |  | TAGCACCATTTGAAAT**TAGTGC** |
|  | RT | GTCGTATCCAGTGCAGGGTCCGAGGTATTCGCACTGGATACGAC**GCACTA** |
|  | Forward | CGCGCGTAGCACCATTTGAA |
|  | Reverse | GTGCAGGGTCCGAGGT |
| lst-miR-n138-1-3p |  | TGCATCCGGCCAAT**TGACTG** |
|  | RT | GTCGTATCCAGTGCAGGGTCCGAGGTATTCGCACTGGATACGAC**CAGTCA** |
|  | Forward | CGCTGCATCCGGCCAAT |
|  | Reverse | GTGCAGGGTCCGAGGT |
| lst-miR-n90-5p |  | TGAGAAGTCAGCGTGGAT**GAGAAG** |
|  | RT | GTCGTATCCAGTGCAGGGTCCGAGGTATTCGCACTGGATACGAC**CTTCTC** |
|  | Forward | CGCGCTGAGAAGTCAGCGT |
|  | Reverse | GTGCAGGGTCCGAGGT |
| lst-miR-n114-5p |  | AATGGCACTGGAAGAATT**CACGGG** |
|  | RT | GTCGTATCCAGTGCAGGGTCCGAGGTATTCGCACTGGATACGAC**CCCGTG** |
|  | Forward | AGCGCAATGGCACTGGAAGAATT |
|  | Reverse | GTGCAGGGTCCGAGGT |
| lst-miR-n210-3p |  | TAAATGCACTATCTGGT**ACGACA** |
|  | RT | GTCGTATCCAGTGCAGGGTCCGAGGTATTCGCACTGGATACGAC**TGTCGT** |
|  | Forward | CGCGCGTAAATGCACTATCTGGT |
|  | Reverse | GTGCAGGGTCCGAGGT |
| lst-miR-n144-3p |  | TGCTGGTTGGGCTGCT**CGGGGG** |
|  | RT | GTCGTATCCAGTGCAGGGTCCGAGGTATTCGCACTGGATACGAC**CCCCCG** |
|  | Forward | AGTGCTGGTTGGGCTGCT |
|  | Reverse | GTGCAGGGTCCGAGGT |
| lst-miR-n141-3p |  | TGCATCTCCAGCTGCCGC**TGCTGC** |
|  | RT | GTCGTATCCAGTGCAGGGTCCGAGGTATTCGCACTGGATACGAC**GCAGCA** |
|  | Forward | AATGCATCTCCAGCTGCCGC |
|  | Reverse | GTGCAGGGTCCGAGGT |
| lst-miR-n199-3p |  | TCCACCGGCAGCTCC**ACTTCC** |
|  | RT | GTCGTATCCAGTGCAGGGTCCGAGGTATTCGCACTGGATACGAC**GGAAGT** |
|  | Forward | GCTAATTCCACCGGCAGCTCC |
|  | Reverse | GTGCAGGGTCCGAGGT |
| lst-miR-n188-3p |  | TACACCTTGTGCGTCT**TGTGCT** |
|  | RT | GTCGTATCCAGTGCAGGGTCCGAGGTATTCGCACTGGATACGAC**AGCACA** |
|  | Forward | CGCGTACACCTTGTGCGTCT |
|  | Reverse | GTGCAGGGTCCGAGGT |
| lst-miR-n88-5p |  | CCTGTGGCACTCTTTG**GTAAGT** |
|  | RT | GTCGTATCCAGTGCAGGGTCCGAGGTATTCGCACTGGATACGAC**ACTTAC** |
|  | Forward | GCGCCTGTGGCACTCTTTG |
|  | Reverse | GTGCAGGGTCCGAGGT |
| lst-miR-n67-5p |  | TGCGCCGTCGGTGGAAA**TACTGC** |
|  | RT | GTCGTATCCAGTGCAGGGTCCGAGGTATTCGCACTGGATACGAC**GCAGTA** |
|  | Forward | TGCGCCGTCGGTGGAAA |
|  | Reverse | GTGCAGGGTCCGAGGT |
| lst-miR-n37-1-5p |  | TATTGCTGGTGATGATG**CTGCGG** |
|  | RT | GTCGTATCCAGTGCAGGGTCCGAGGTATTCGCACTGGATACGAC**CCGCAG** |
|  | Forward | CGCGCGTATTGCTGGTGATGATG |
|  | Reverse | GTGCAGGGTCCGAGGT |
| *5.8S* rRNA (106 bp) | Forward | TGCGTGACAAACTGTGAACT |
|  | Reverse | CCGACCCTCAGACAGACAT |
| *β*-actin (194 bp) | Forward | GCCCATCTACGAAGGTTAC |
|  | Reverse | CCATTTCCTGTTCGAAGTCCAG |
