# Supplementary material for: Identification and Characterization of MicroRNAs in Small Brown Planthopper (Laodephax striatellus) by Next-Generation Sequencing
Source: PLoS One. 2014 Jul 24;9(7):e103041. doi: 10.1371/journal.pone.0103041 (PMC4109989; doi:10.1371/journal.pone.0103041)
Supplement: Table S8 — The outer and inner primer sequences of RLM-5′RACE. (DOCX) [file pone.0103041.s010.docx]

**Table S8. The outer and inner primer sequences of RLM-5’RACE.**

| miRNA ID | mRNA gene | Primers | Sequences | |
| --- | --- | --- | --- | --- |
|  |  | 5’RACE forward outer primer | CATGGCTACATGCTGACAGCCTA |  |
|  |  | 5’RACE forward inner primer | CGCGGATCCACAGCCTACTGATGATCAGTCGATG | |
| lst-miR-981-3p | Cluster1528-Consensus1 | Gene reverse outer primer | CGTGTGTTGTCTGGTTCATCGTCT | |
|  |  | Gene reverse inner primer | CGGTTCTTCTGATGCACGACGTAGG | |
| lst-miR-n59-5p | Cluster2118-Consensus1 | Gene reverse outer primer | GCTCATGCCCGTTGACAGAATCA | |
|  |  | Gene reverse inner primer | ATGCCAGGTCCTCCACCCGTCT | |
